# Reciprocal regulation of GPNMB/HIF-1α for Inhibition of neuronal ferroptosis in delayed encephalopathy after acute carbon monoxide poisoning

**DOI:** 10.1186/s40478-025-02069-x

**Published:** 2025-07-14

**Authors:** Zuolong Liu, Lanyue Sun, Nan Gao, Wei Li, Li Pang

**Affiliations:** 1 Department of Emergency, The First Hospital of Jilin University, No. 1 Xinmin Road, Changchun, Jilin Province 130021 P.R. China; 2 Medical Quality Control Office, The Third Affiliated Hospital of Changchun, University of Chinese Medicine, Changchun, Jilin Province 130118 P.R. China

**Keywords:** GPNMB, HIF-1α, STAT3, Ferroptosis, DEACMP

## Abstract

Delayed encephalopathy after acute carbon monoxide poisoning (DEACMP) is the most common complication after acute carbon monoxide (CO) poisoning. However, the pathogenesis of DEACMP remains ambiguous. The neuroprotective role of GPNMB has been observed in amyotrophic lateral sclerosis and Parkinson’s disease. GPNMB was elevated in the brain tissues of DEACMP rats, while its function in DEACMP remains unclear. In this study, a CO poisoning rat model and oxygen-glucose deprivation (OGD)-treated PC-12 cells were established as an in vivo and in vitro DEACMP model, respectively. The ferroptosis inhibitor Ferrostatin-1 (Fer-1) ameliorated cognitive impairment, inflammation and oxidative stress of rats with DEACMP as assessed by Morris Water Maze test, ELISA assay and commercial kits of oxidative markers. Immunofluorescence, qRT-PCR or western blot showed that GPNMB was elevated in CA1 hippocampal tissues of CO-poisoned rats. Additionally, TUNEL staining, ELISA assay and western blot revealed that GPNMB rescued OGD-induced cell apoptosis, inflammation and ferroptosis in PC-12 cells. Mechanistical study showed that STAT3 was a transcriptional activator of GPNMB as detected by luciferase and ChIP assays, and co-immunoprecipitation and immunofluorescence staining revealed that GPNMB stabilized HIF-1α by direct binding. Functionally, GPNMB protected against OGD-induced impairments via inducing HIF-1α. Furthermore, GPNMB attenuated cognitive impairment, oxidative stress and neuronal ferroptosis of rats with DEACMP. In conclusion, GPNMB/HIF-1α exhibited neuroprotective effects via suppressing ferroptosis in DEACMP.

**Supplementary Information:**

The online version contains supplementary material available at 10.1186/s40478-025-02069-x.

## Introduction

Carbon monoxide (CO) poisoning continues to be a common occupational hazard worldwide, with its incidence in China showing a marked increase over the past two decades [1, 2]. Delayed encephalopathy after acute carbon monoxide poisoning (DEACMP) is the most frequently diagnosed complication after acute CO poisoning, affecting 20 to 40% of patients [3]. DEACMP patients exhibit acute symptoms, such as motor impairment, cognitive and extrapyramidal system dysfunction, as well as consciousness disturbance after a symptom-free interval [4]. Unfortunately, the clinical treatment of DEACMP remains symptomatic due to the limited understanding of DEACMP pathogenesis. Unraveling the regulatory mechanism underlying DEACMP development will provide novel insight into DEACMP therapeutic strategy.

Ferroptosis is a new form of regulated cell death (RCD) characterized with iron-dependent accumulation of lipid reactive oxygen species (ROS) [5]. Emerging evidence supports that elevated lipid peroxidation is associated with DEACMP pathogenesis [6–8]. Recent research has reported that ferroptosis inhibition protects against acute carbon monoxide poisoning (ACMP)-induced white matter injury [7]. Intriguingly, CO overexposure upregulates ROS production which interacts with polyunsaturated fatty acid in CNS to drive lipid peroxidation [9]. These findings suggest that ferroptosis might play an important role in DEACMP pathogenesis. In recent years, a number of studies have reported that targeting ferroptosis by lipophilic antioxidants or iron chelators ameliorates cognitive dysfunction in various neurological diseases [10–12]suggesting potential therapeutic benefits for DEACMP patients. In addition, recent studies have illustrated that HIF-1α suppresses ferroptosis in brain injury [13, 14]. For instance, HIF-1α inhibits ferroptosis in brain neurons and improves cerebral ischemic/reperfusion injury through FPN1/Nrf2/HO-1 axis [14]. However, whether HIF-1α participates in the regulation of ferroptosis in DEACMP remains undefined.

Glycoprotein non-metastatic melanoma protein B (GPNMB), a transmembrane glycoprotein, has been recognized as a tumor promoter in different cancers [15]. Beyond its well-studied oncogenic role, GPNMB is ubiquitously expressed in microglia, macrophages, astrocytes and neurons [16]. Recently, emerging evidence has supported the neuroprotective roles of GPNMB in multiple neurological diseases [17–19]. For instance, GPNMB is recognized as a neuroprotective factor that mitigates motor neuron degeneration in amyotrophic lateral sclerosis (ALS) [17]. Moreover, GPNMB ameliorates astrocyte-mediated inflammation through CD44 receptor in Parkinson’s disease (PD) [19]. Growing evidence suggests that ferroptosis plays a pivotal role in inflammatory response [20, 21]. Given that ferroptosis is known to trigger the release of pro-inflammatory cytokines IL-18 and IL-1β [21]investigating whether GPNMB alleviates neuroinflammation via suppressing ferroptosis merits in-depth investigation. However, the role of GPNMB in DEACMP remains uninvestigated. The preliminary expression analysis showed remarkable elevation of GPNMB in the brain tissues of DEACMP rats. Considering the critical functions of GPNMB in lipid metabolism, inflammation and ROS production [19, 22]it was hypothesized that GPNMB might exhibit a neuroprotective role in DEACMP by modulating ferroptosis.

In line with our hypothesis, this study reported that the ferroptosis inhibitor Ferrostatin-1 (Fer-1) attenuated cognitive impairment, neuroinflammation and oxidative stress in DEACMP rat model. GPNMB was elevated in CA1 hippocampal tissues of CO-poisoned rats. In OGD-treated PC-12 cells, GPNMB ameliorated DNA fragmentation, inflammation and ferroptosis. Bioinformatics analysis predicted the potential binding between STAT3 and *GPNMB*, as well as between GPNMB and HIF-1α. Mechanistically, STAT3 was identified as a transcriptional activator of GPNMB, and GPNMB stabilized HIF-1α through direct binding. In vitro functional experiments showed that GPNMB protected against OGD-induced impairments via modulating HIF-1α. In addition, in vivo study further revealed that GPNMB improved cognitive function, reduced oxidative stress and suppressed neuronal ferroptosis in rats with DEACMP.

## Materials and methods

### Animals and DEACMP rat model

All animal studies were approved by the Ethics Committee of The First Hospital of Jilin University (No. 2023-0007). All the animal procedures were performed in accordance with the Guiding Principles in the Use and Care of Animals. Male Sprague-Dawley rats (~ 220–240 g b.w., *N* = 12 per group) were from The First Hospital of Jilin University. Rats were housed in controlled condition with alternating 12 h light and dark cycles. DEACMP rat model was established as previous described [23]. Briefly, rats were exposed to 1000 ppm CO for 40 min and additional 3000 ppm CO for 20 min until the unconsciousness state. Rats were then replaced to the chamber with normal condition air to regain consciousness. Rats in Sham group were placed in the chamber with normal condition air. For the treatment of ferroptosis inhibitor Ferrostain-1 (Fer-1), rats were administered with 10 mg/kg Fer-1 (SML0583, Sigma-Aldrich, St. Louis, MO, USA) intraperitoneally after CO exposure as described [7]. After CO poisoning, in vivo overexpression or knockdown of GPNMB were administered with intracerebroventricular (ICV) injection with GPNMB or sh-GPNMB (0.25 µL; 4 × 10^6^ TU/µL; GenePharma, Shanghai, China). Briefly, rats were anesthetized by intraperitoneal injection of 50 mg/kg phenobarbital and fixed on the stereotaxic apparatus. The left striatum was targeted using stereotaxic coordinates (0–0.5 mm anterior to bregma, 2.5–3 mm lateral to midline, and 5–6 mm ventral to dura) as described [24]. Following coordinate calibration, a cranial burr hole was created using a dental micro-drill and a guide cannula was implanted. The injection cannulas were connected to Hamilton syringes and inserted into the guide cannulas. GPNMB or sh-GPNMB was injected into the lateral ventricles at a speed of 0.5 µL/min using a syringe pump (KD Scientific, Holliston, MA, USA) once a day for 2 days [24, 25]. Rats were maintained at 37 ℃ using a heating pad during the whole experiment, and hourly vital sign monitoring continued throughout post-operative recovery.

## Morris water maze (MWM) test

The memory and learning performance of rats were assessed using MWM apparatus (Zhenghua Biologicals, Anhui, China) on 1–5 days post-CO exposure [26]. The MWM test was conducted in a white circular pool with a diameter of 150 cm and a depth of 30 cm. A platform (9 cm in diameter) was placed 1 cm beneath the opaque water in the middle of the target quadrant. Rats were randomly placed in the pool at one of four quadrants, and allowed to climb onto the platform within the maximum time of 1 min. If they failed to find the platform in 1 min, they were guided to the platform and remained there for 15 s. On day 1–5, rats were placed in the entry site of the pool, and the escape latency, swimming distance and speed were recorded and automatically calculated using Smart digital tracking system (Panlab, Barcelona, Spain).

## Histopathological analysis

The brains were fixed with 10% formalin for 48 h, and placed in 10% formalin/30% sucrose for cryoprotection. The hippocampal tissues were cut on cryostat. Frozen Sect. (5 μm-thickness) were stained with hematoxylin and eosin (H&E, Sigma-Aldrich) or cresyl violet solution for Nissl staining (Sigma-Aldrich). Images were acquired under a microscopy (Nikon, Tokyo, Japan). Nissl body number was quantified as previously described [27]. Single neuron was captured, and Nissl bodies (blue-purple dots) were defined using SigmaScan 4.0 Software (Systat Software, San Jose, CA, USA). The number of Nissl body was quantified using Image J software 1.53o (National Institute of Health, http://rsb.info.nih.gov/ij, last accessed May 28, 2023).

## Iron staining

The iron accumulation in rat hippocampus or PC-12 cells was measured by FerroOrange staining [28]. Briefly, sections or PC-12 cells were stained with FerroOrange Probe (1 µM, F374, DOJINDO, Japan) at 37 ℃ for 30 min. Images were acquired under a confocal microscope (Nikon). Iron-positive cells were counted on a total of four randomly-selected microscopic fields as described [29].

## Immunohistochemistry (IHC) analysis

The frozen sections were permeabilized with 0.1% Triton X-100, and blocked with 1% BSA. Sections were then stained with anti-GPX4 (1:100, ab125066, Abcam, Cambridge, UK), anti-GPNMB (1:100, ab175427, Abcam) or anti-STAT3 (1:200, ab68153, Abcam) antibody at 4 ℃ overnight, and followed by the incubation with secondary antibody-HRP (31460, Invitrogen, Carlsbad, CA, USA). Signals were detected using DAB substrate (Pierce, Rockford, IL, USA). For the immunofluorescent detection of GPNMB, Alexa Fluor 488-conjugated goat anti-mouse antibody (1:1000, A11001, Invitrogen) was used. Quantification of IHC staining was using Image J Version 1.53o (National Institute of Health) as described [30]. At least three sections per rat and five randomly-selected microscopic fields per section were selected for GPX4- or GPNMB-positive cell counting. The numbers of GPX4- or GPNMB-positive cells in 400X fields were counted, and the average of five microscopic fields in each group was presented.

### Cell culture, transfection and oxygen-glucose deprivation (OGD) cell model

PC-12 cells were from ATCC (Manassas, VA, USA) and grown in DMEM containing 10% FBS (Gibco, Grand Island, NY, USA) at 37 ℃/5% CO_2_. For OGD treatment, PC-12 cells were maintained in glucose-free DMEM in an oxygen-free condition (95% N_2_ and 5% CO_2_) for 4 h. For nerve growth factor (NGF) treatment, PC-12 cells were treated with 100 ng/mL NGF (Sigma-Aldrich) for 3 days as described [31]. Differentiated PC-12 cells were used in the subsequent experiments. Cells were then replaced in normal DMEM supplemented with 10% FBS, and maintained under normoxic condition for 24 h. According to the NCBI access GPNMB gene sequence (https://www.ncbi.nlm.nih.gov/gene/?term=GPNMB, last accessed April 13, 2021), three shRNAs against rat GPNMB (designed by GenePharma), namely sh-GPNMB#1, sh-GPNMB#2 and sh-GPNMB#3, were used in the preliminary experiments. sh-GPNMB#2 with higher knockdown efficacy was selected and packaged into the AAV2/9-CMV expression vector, purchased from Shanghai TaiTu Biotechnology Co., Ltd., which was prepared for the subsequent experiments. In the GPNMB gene overexpression experiment for rats or PC-12 cells, recombinant lentiviral vector backbone pCDH-CMV-MCS-EF1-CopGFP-T2A Puro carrying GPNMB or its negative control vector was designed and assembled by Servicebio Company (Wuhan, China). siSTAT3, siHIF-1α or corresponding controls were purchased from Genechem (Shanghai, China). The sequences referring to these plasmids were shown in Table 1. The full-length of rat STAT3 were cloned into pcDNA3.1 vector. PC-12 cells were transfected with siRNA or overexpression plasmid using Lipofectamine 3000 (Invitrogen). At 48 h post-transfection, PC12 cells were subjected to OGD/R treatment (OGD, 4 h/R, 24 h) as described [32].

**Table 1:** ShRNA and SiRNA sequences

shRNA	Sequence
rat GPNMB#1	5’-CACCATTGTTGGAGACAAACAGGCCACGAATGGCCTGTTTGTCTCCAACAA-3’
rat GPNMB#2	5’-CACCAATGTCACTCAGCTCCATGGATCGAAATCCATGGAGCTGAGTGACA − 3’
rat GPNMB#3	5’- CACCGAAATTCACACAGTACGTGCCGCGAACGGCACGTACTGTGTGAA − 3’
**siRNA**	**Sequence**
si-rat STAT3	5’-GGAGCAGCACCUUCAGGAUdTdT-3’
si-rat HIF-1α	5’-AAAGAGGTGGATATGTCTGGG-3’

### TUNEL staining

DNA fragmentation was assessed using TUNEL Assay Kit-FITC (ab66108, Abcam) or TUNEL Assay-Kit-HRP-DAB (ab206386, Abcam). Briefly, PC-12 cells were fixed with 1% paraformaldehyde (PFA) and placed in ice-cold 70% ethanol. After rinsing, cells were then incubated with Staining Solution at 37 ℃ for 2 h, followed by the staining with DAPI. Images were acquired using a confocal microscope (Nikon). For the staining of frozen section, similar procedure was conducted with DAB development and counterstain with Methy Green. Images were acquired under a microscope (Nikon).

### ELISA assay

The levels of IL-6 (BMS625, Invitrogen), TNF-α (BMS622, Invitrogen) and IL-1β (BMS630, Invitrogen) in rat hippocampus or cell culture medium of PC-12 cells was quantified using commercial ELISA kits. In brief, rat brain tissues or cell culture supernatant was collected and subjected to ELISA assay according to the manufacturer’s protocol. The level of 8-OHdG in CA1 hippocampal tissues was measured using 8-OHdG ELISA kit (CSB-E10140h, Cusabio, Wuhan, China). A450 was measured using a microplate reader (Bio-Rad, Hercules, CA, USA).

### Measurement of the carbonyl content, MDA, GSH, iron content or ROS

Protein carbonyl content was assessed using Protein Carbonyl Content Assay Kit (ab126287, Abcam). Briefly, homogenized rat hippocampal tissues were incubated with DNPH for 10 min, followed by the incubation with TCA. The pellet was rinsed with cold acetone and resolubilized in Guanidine solution. A375 was measured using a microplate reader (Bio-Rad).

MDA was quantified using MDA Assay Kit (ab118970, Abcam). In brief, homogenized rat hippocampal tissues, PC-12 cells and standards were incubated with TBA reagent at 95 °C for 1 h. A532 was measured using a microplate reader (Bio-Rad).

GSH was measured using Reduced Glutathione Assay Kit (A006-2, Nanjing Jiancheng, Nanjing, China). Briefly, rat hippocampal tissues or PC-12 cells were lysed, and incubated with Reagent 2 and 3 for 5 min. A405 was measured using a microplate reader (Bio-Rad).

The total iron content was quantified using Iron Assay Kit (MAK025, Sigma-Aldrich). Briefly, PC-12 cells were homogenized in Iron Assay buffer. The reaction mixture was incubated for 30 min, followed by the incubation with Iron Probe for 1 h. A593 was measured using a microplate reader (Bio-Rad).

Cellular ROS was measured using Cellular ROS Assay Kit (ab113851, Abcam). Briefly, PC-12 cells were rinsed with 1X Buffer and stained with 2′,7′-dichlorodi-hydrofuorescein diacetate (DCFH-DA) solution for 45 min. The fluorescence was measured at Ex/Em 485/535 nm using a microplate reader (Bio-Rad).

### Dual luciferase reporter assay

The promoter region of GPNMB was cloned into pGL-3 vector. The luciferase construct and siSTAT3/STAT3 overexpression plasmid were co-transfected into PC-12 cells using Lipofectamine 3000 reagent (Invitrogen). The relative luciferase activity was quantified using Dual Luciferase Reporter System (Promega, Madison, WI, USA) at 48 h post-transfection. The ratio of luciferase signal from Firefly/Renilla was normalized to siNC or Vector control.

### Chromatin immunohistochemistry (ChIP) assay

ChIP assay was conducted using Pierce Agarose ChIP Assay Kit (26156, Pierce). In brief, PC-12 cells were cross-linked with 1% formaldehyde, and the chromatin was digested with MNase. The chromatin fragments were then incubated with anti-STAT3 (2 µg, MA1-13042, Invitrogen) antibody or normal IgG at 4 °C overnight, and the protein-DNA complexes were enriched by Protein A/G agarose. The immunoprecipitated DNA was purified and analyzed by qRT-PCR.

### RNA isolation and qRT-PCR

Total RNA was extracted from PC-12 cells or hippocampal tissues using Trizol (Invitrogen). qRT-PCR was performed using SYBR Green quantitative RT-qPCR kit (Sigma-Aldrich) in ABI7500 Real-Time PCR System (ABI, Foster City, CA, USA). Briefly, 5 ng RNA template was addition into the master mix containing SYBR Green Taq ReadyMix, MgCl_2_, M-MLV RT and primers, followed by thermal cycling according to the manufacturer’s protocol. The relative expression of target gene was calculated using 2 ^–ΔΔCT^ method. The primers used in this experiment were listed in Table 2.

**Table 2:** The sequences of primers used in this study

Primer	Sequence
GPNMB sense	5’-TCCTGGTGGATGGGACTAGG-3’
GPNMB anti-sense	5’-CCCCCAAACTCCAGTCAAGG-3’
GPNMB sense (For ChIP)	5’-AGCACCCAAGGGACACAAAA-3’
GPNMB anti-sense (For ChIP)	5’-ACTCCCTAGTCCCATCCACC-3’
STAT3 sense	5’-AGAGGCGGCAGCAGATAGC-3’
STAT3 anti-sense	5’-TTGTTGGCGGGTCTGAAGTT-3’
HIF-1α sense	5’-CAGCAGACCCAGTTACAGAA-3’
HIF-1α anti-sense	5’-CAGCAGACCCAGTTACAGAA-3’
β-actin sense	5’-CTTAGTTGCGTTACACCCTTTCTTG-3’
β-actin anti-sense	5’-CTGTCACCTTCACCGTTCCAGTTT-3’

### Protein extraction and Western blot

Cell or tissue lysates were prepared using RIPA lysis buffer (Pierce). Proteins were quantified using BCA Protein Assay Kit (Pierce), and separated by gel electrophoresis. Proteins were then transferred onto PVDF membrane (Pierce) and blocked with 5% non-fat milk. The blots were incubated with anti-rat SYP (1;5000, ab32127, Abcam), anti-rat PSD95 (1:1000, ab18258, Abcam), anti-rat GPNMB (1:1000, PA5-89716, Invitrogen), anti-rat GPX4 (1:1000, ab125066, Abcam), anti-rat STAT3 (1:1000, ab68153, Abcam), anti-rat p-STAT3 (1:1000, ab30647, Abcam), anti-rat HIF-1α (1:1000, ab179483, Abcam), anti-rat ubiquitin (1:1000, ab140601, Abcam), anti-rat GAPDH (1:2000, ab8245, Abcam) or anti-rat β-actin (1:2000, ab8226, Abcam) antibody at 4℃ overnight, followed by the incubation with secondary antibody-HRP (31460 or 31430, Invitrogen). Signals were detected using SuperSignal Pico Plus Kit (Pierce).

### Co-immunoprecipitation (co-IP)

PC-12 cells were lysed with IP lysis buffer (Pierce). Protein lysates were incubated with anti-GPNMB (1:30, ab188222, Abcam), anti-HIF-1α (1:30, PA1-16601, Invitrogen) antibody or normal IgG at 4℃ overnight. The protein complexes were enriched by Protein A/G magnetic beads (Pierce), and the elutes were analyzed by western blot. Normal IgG served as a negative control, and whole cell lysates were used as an input control.

### Immunofluorescence (IF)

PC-12 cells were fixed with 4% PFA and permeabilized with 0.1% Triton X-100. After blocking with 1% BSA, cells were then incubated with anti-HIF-1α (1:500, ab179483, Abcam) antibody at 4℃ overnight. This is followed by the incubation with Alexa Fluor 555-conjugated secondary antibody (1:500, A-21428, Invitrogen) and Alexa Fluor 488-conjugated anti-GPNMB (1:50, ab284639, Abcam) antibody. Images were acquired using a confocal microscope (Nikon).

### Statistical analysis

Experiments were performed in triplicate and error bars represent standard deviation (SD) of a triplicate set of experiments. Data were analyzed using SPSS version 22.0. One-way ANOVA followed by *Bonferroni’s multiple comparison tests* or two-tailed Student’s *t*-test was used to assess the differences among multiple groups or between two groups, respectively. For MWM tests, the results were analyzed by repeated measures two-way ANOVA followed by *Bonferroni post hoc analysis* [33, 34]. *P* < 0.05 was considered statistically significant.

## Results

### Fer-1 attenuates cognitive impairment of rats with DEACMP

**Chart. 1:**
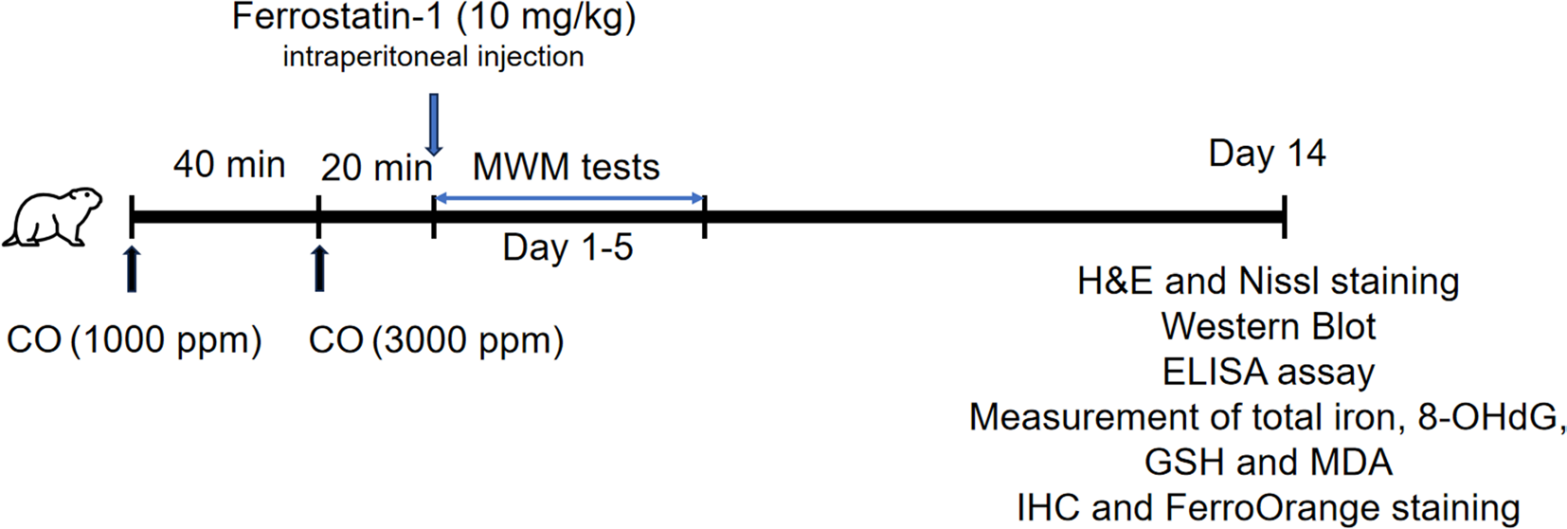
A timeline diagram for summarizing animal experimental design

**Fig. 1:**
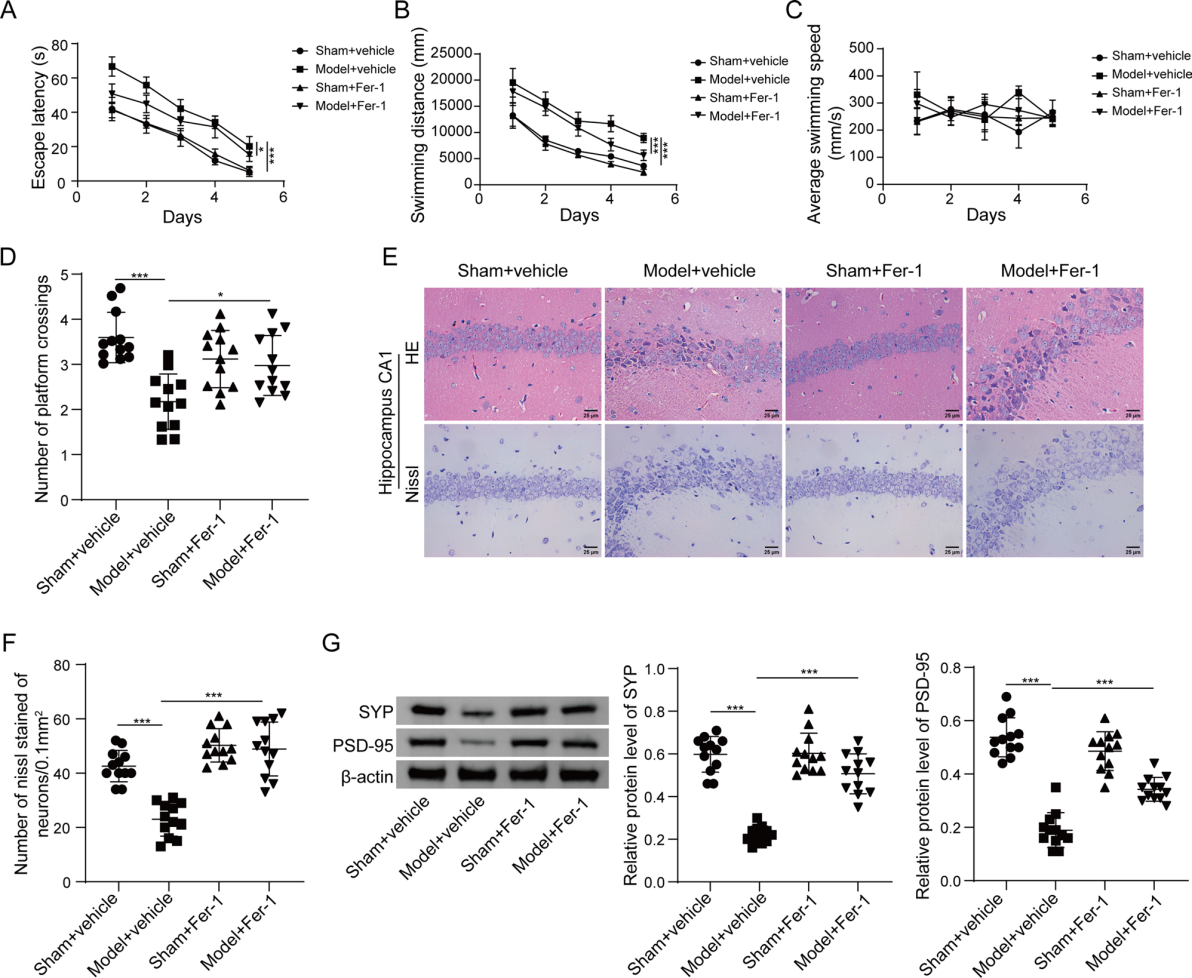
Fer-1 attenuates cognitive impairment of rats with DEACMP. (**A**) Escape latency was evaluated by MWM tests. (**B**) Swimming distance was evaluated by MWM tests. (**C**) Average swimming speed was assessed by MWM tests. (**D**) The number of platform crossing was recorded. For each day of training, data were averaged across five daily trials. (**E**-**F**) The histological changes in rat CA1 hippocampal tissues were assessed by H&E staining, and the morphology of hippocampal neurons was detected by Nissl staining. Scale bar, 25 μm. (**G**) The protein levels of SYP and PSD95 in rat CA1 hippocampal tissues were detected by western blot. *N* = 12 per group. (**A**-**D**) were analyzed by repeated measures two-way ANOVA followed by *Bonferroni post hoc analysis*; (**F**-**G**) were analyzed by one-way ANOVA followed by *Bonferroni’s multiple comparison tests*. *, *P* < 0.05; **, *P* < 0.01; ***, *P* < 0.001

To delineate the role of ferroptosis in DEACMP pathogenesis, the ferroptosis inhibitor Fer-1 was administered to the CO-poisoned and sham rats (details shown in Chart 1 for our experimental design). MWM tests showed that CO-poisoned rats exhibited prolonged escape latency, longer swimming distance and reduced swimming speed, compared with that of sham rats (Fig. 1A-C), all of which were attenuated by Fer-1 treatment (Fig. 1A-C). The memory of rats was evaluated by measuring the numbers of platform crossing. As presented in Fig. 1D, the platform crossing number was remarkably decreased in CO-poisoned rats, while Fer-1 ameliorated CO poisoning-induced memory impairment. In addition, H&E staining further showed well-organized hippocampal neurons in sham rats, whereas CO-poisoned rats exhibited neuronal degeneration, disintegration and necrosis in CA1 hippocampal tissues (Fig. 1E). Nissl staining also revealed that the number of Nissl body in model rats was much more than that in sham group, while a partial rebound of Nissl body number was found in Model + Fer-1 group (Fig. 1E-F). Western blot showed that the synaptic proteins SYP and PSD95 were downregulated in CA1 hippocampal tissues upon CO exposure, while Fer-1 restored their expression (Fig. 1G). These data indicate that Fer-1 ameliorates CO poisoning**-**induced cognitive impairment in rats.

### Fer-1 attenuates inflammatory impairment and oxidative stress of rats with DEACMP

**Fig. 2:**
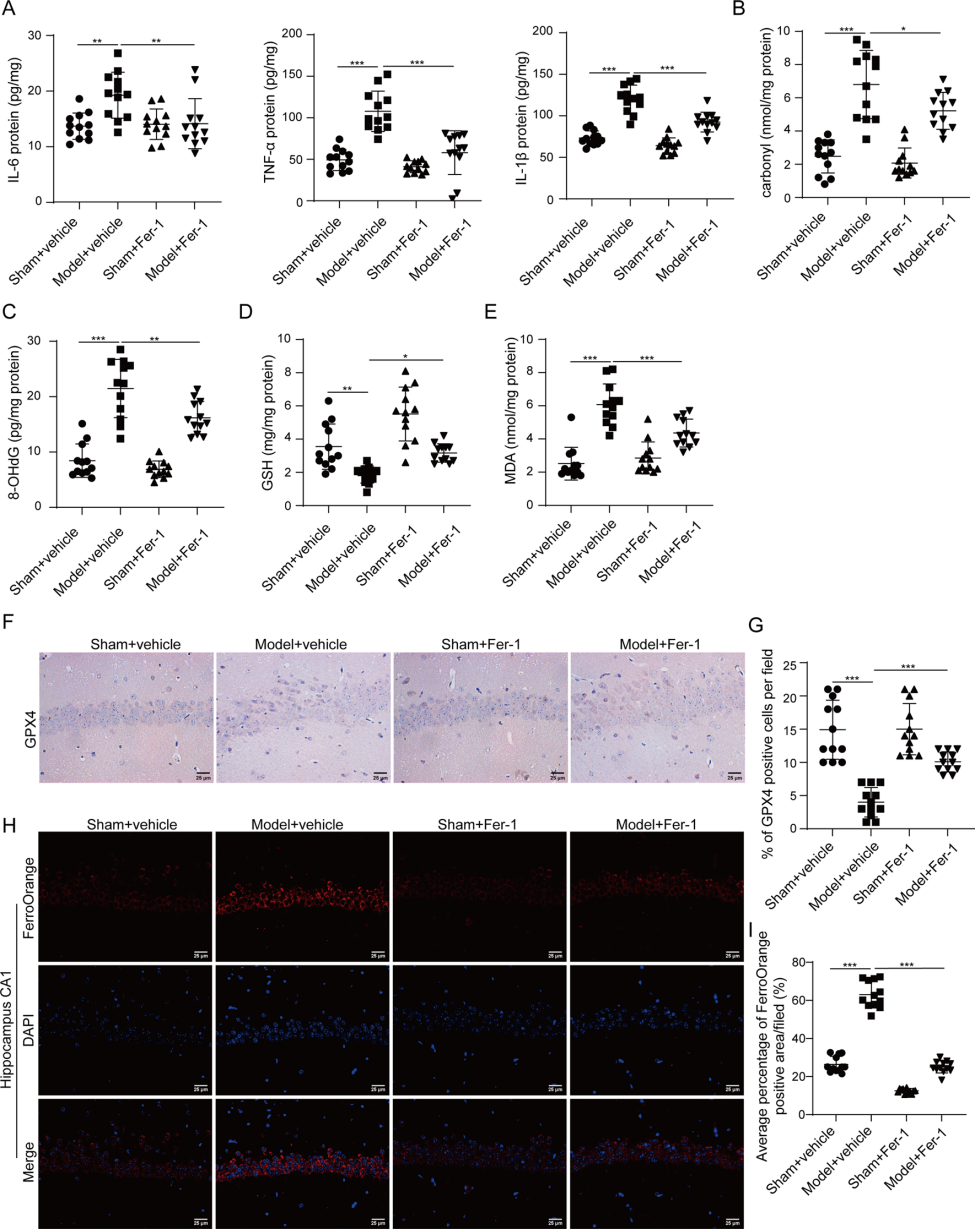
Fer-1 attenuates inflammatory impairment and oxidative stress of rats with DEACMP. (**A**) The levels of pro-inflammatory cytokines in hippocampus CA1 area were measured by ELISA assay. (**B**-**C**) The levels of protein carbonyl content and 8-OHdG in hippocampus CA1 area were assessed using commercial kits. (**D**-**E**) The levels of GSH and MDA in hippocampus CA1 area were measured using commercial kits. (**F**-**I**) Iron accumulation in hippocampus CA1 area was detected by FerroOrange staining, and the immunoreactivity of GPX4 in hippocampus CA1 area was detected by IHC analysis. Red, FerroOrange; Blue, DAPI. Scale bar, 25 μm. *N* = 12 per group. (**A**-**E**, **G**-**I**) were analyzed by one-way ANOVA followed by *Bonferroni’s multiple comparison tests*. *, *P* < 0.05; **, *P* < 0.01; ***, *P* < 0.001

The researchers next sought to investigate the effects of Fer-1 on inflammation and oxidation in DECAMP. ELISA assay showed the elevations of pro-inflammatory cytokines IL-6, TNF-α and IL-1β within CA1 hippocampal tissues of model rats, while Fer-1 exerted protective effects on the levels of these cytokines (Fig. 2A). Additionally, the markers of oxidative stress, namely carbonyl content and 8-OHdG, were induced by CO poisoning in CA1 hippocampal tissues, whereas Fer-1 treatment, at least in part, rescued CO poisoning-induced oxidative stress (Fig. 2B-C). Moreover, the reduction of GSH and the induction of MDA were also observed in CA1 hippocampal tissues of model rats, and Fer-1 partially counteracted the effects of CO poisoning on GSH and MDA levels (Fig. 2D-E), implicating lipid peroxidation in this pathogenic process. Furthermore, iron accumulation was markedly higher in CA1 hippocampal tissues of model rats, along with decrease of anti-oxidant enzyme GPX4, in comparison with that of sham rats. By contrast, Fer-1 partially abrogated CO poisoning-induced iron accumulation and GPX4 reduction in hippocampus CA1 area (Fig. 2F-I). Collectively, these findings suggest that Fer-1 ameliorates inflammation and oxidation in rats with DEACMP.

### Expression pattern of GPNMB in rats with DECAMP

**Fig. 3:**
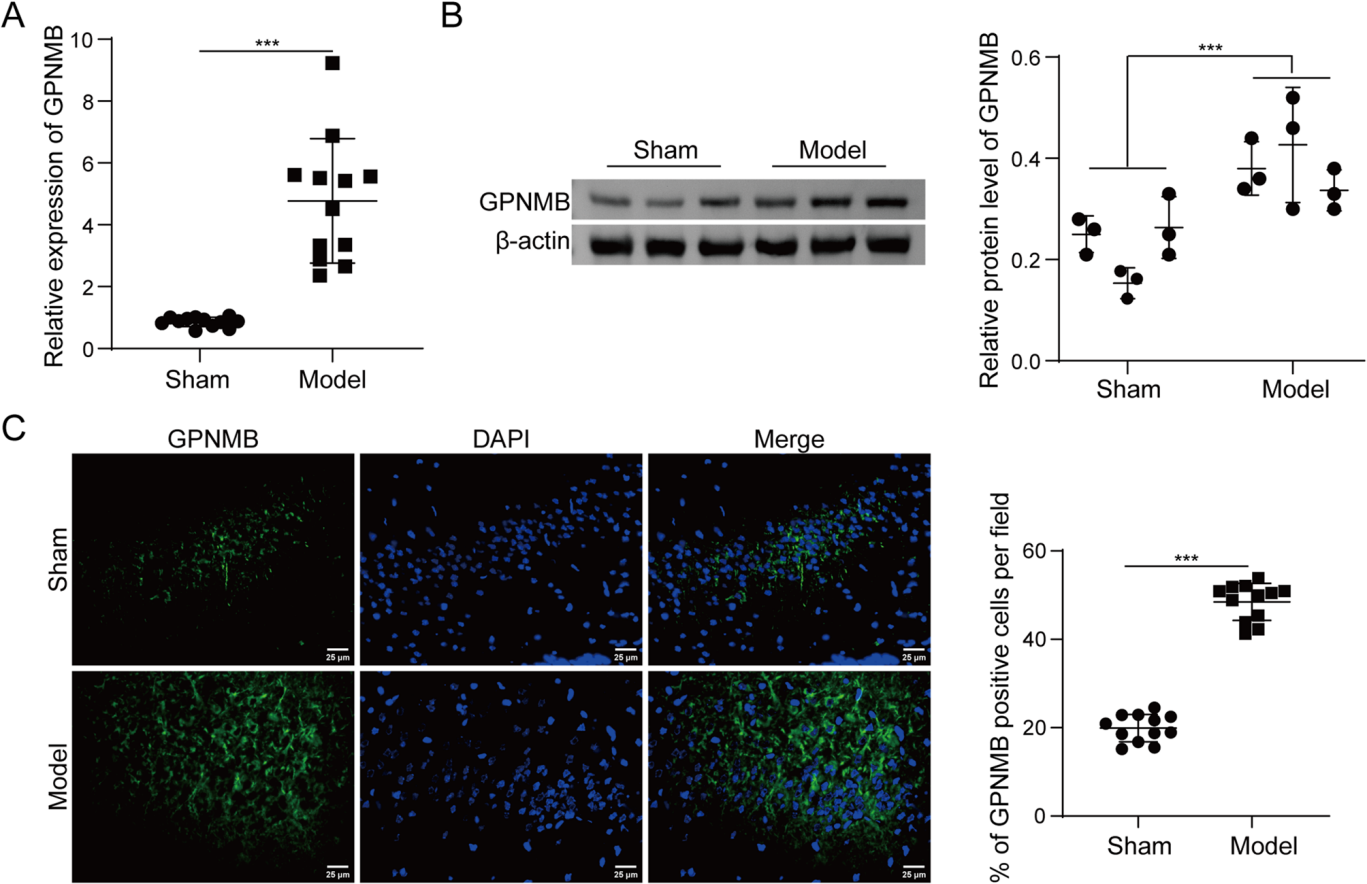
Expression pattern of GPNMB in rats with DECAMP. (**A**) The mRNA level of GPNMB in hippocampus CA1 area was detected by qRT-PCR. (**B**) The protein level of GPNMB in in hippocampus CA1 area was detected by western blot. *N* = 3. (**C**) Representative image of GPNMB staining in hippocampus CA1 area IF staining with quantitative analysis. Green, GPNMB; Blue, DAPI. Scale bar, 25 μm. *N* = 12 per group. (**A**-**C**) were analyzed by two tailed Student’s *t*-test. ***, *P* < 0.001

To unravel the role of GPNMB in DECAMP, the expression of GPNMB was examined in sham and model rats. As presented in Fig. 3A-B, the mRNA and protein levels of GPNMB were dramatically upregulated in CA1 hippocampal tissues of CO-poisoned rats as detected by qRT-PCR and western blot, respectively. IF analysis further revealed that the number of GPNMB-positive cells was markedly increased in hippocampus CA1 area derived from model rats (Fig. 3C). These data indicate the elevation of GPNMB in rats with DEACMP.

### Role of GPNMB on ferroptosis in OGD-treated PC-12 cells

**Fig. 4:**
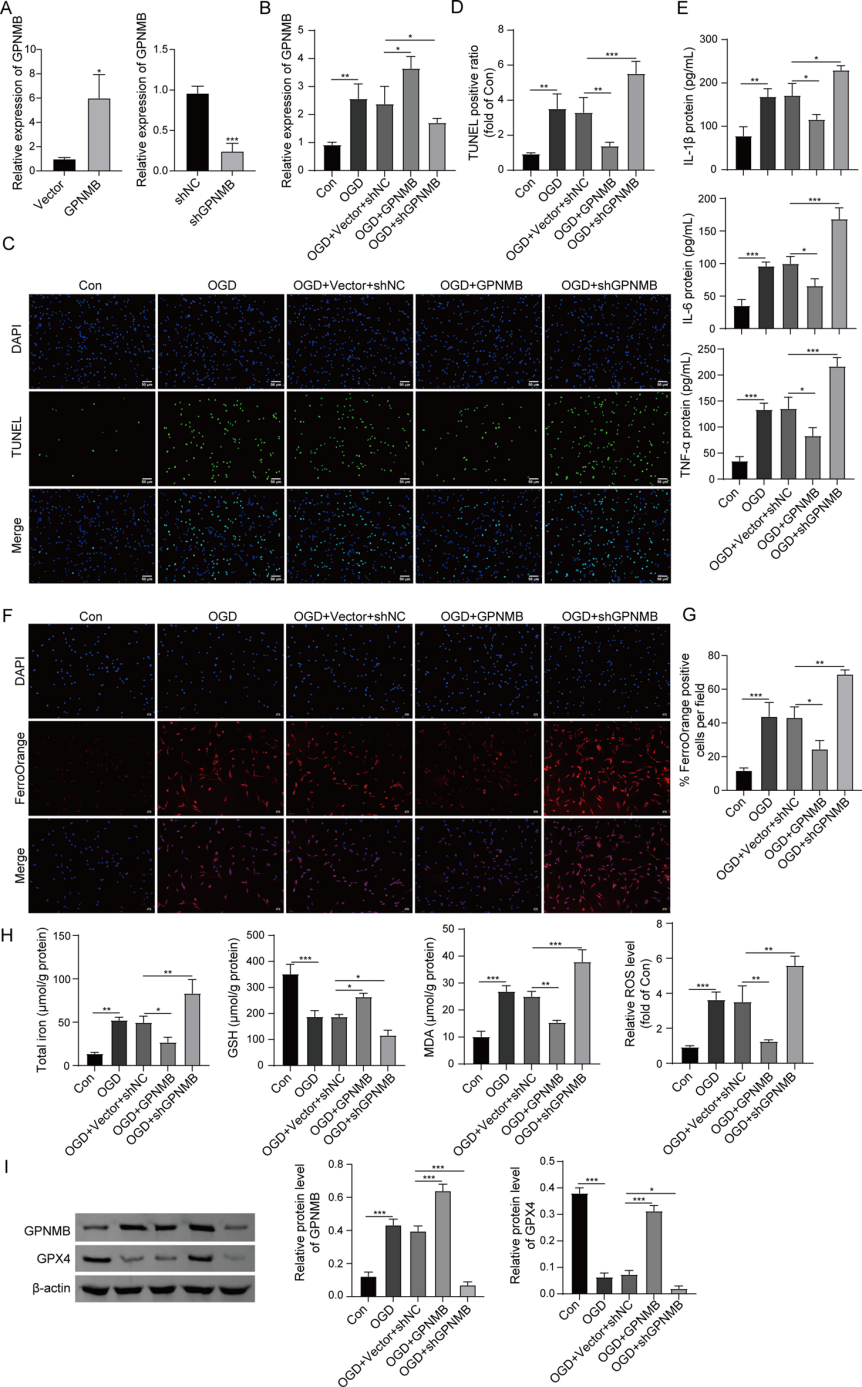
Role of GPNMB on ferroptosis in OGD-treated PC-12 cells. (A-B) The mRNA level of GPNMB in PC-12 cells was detected by qRT-PCR. (**C**-**D**) Cell apoptosis was assessed by TUNEL assay with quantitative analysis. Green, TUNEL; Blue, DAPI. Scale bar, 50 μm. (**E**) The secretion of pro-inflammatory cytokines in cell culture supernatant were measured by ELISA assay. (**F**-**G**) Iron accumulation in OGD-treated PC-12 cells was detected by FerroOrange staining with quantitative analysis. Red, FerroOrange; Blue, DAPI. Scale bar, 50 μm. (**H**) The levels of total iron, GSH, MDA and ROS in PC-12 cells were measured using commercial kits. (**I**) The protein levels of GPNMB and GPX4 were detected by western blot. Experiments were performed in triplicate and error bars represent SD of a triplicate set of experiments. Data are shown as mean ± SD. (**A**) were analyzed by two tailed Student’s *t*-test; (**B**, **D**-**E**, **G**-**I**) were analyzed by one-way ANOVA followed by *Bonferroni’s multiple comparison tests*. *, *P* < 0.05; **, *P* < 0.01; ***, *P* < 0.001

To further investigate whether GPNMB modulated DECAMP pathogenesis through ferroptosis regulation, gain- and loss-of-function experiments were carried out. As anticipated, successful GPNMB overexpression and knockdown were confirmed at both mRNA and protein levels (Fig. 4A). The results of preliminary experiments showed that transfection of shGPNMB#1, shGPNMB#2 or shGPNMB#3 reduced GPNMB mRNA and protein levels, and shGPNMB#2 exhibited the highest knockdown efficiency (Fig.S1A-B). In addition, OGD-induced GPNMB was potentiated in GPNMB-overexpressing cells, while it was counteracted by GPNMB knockdown (Fig. 4B). TUNEL assay showed that OGD-triggered apoptosis was rescued by GPNMB overexpression, whereas more severe apoptosis was observed in GPNMB-knockdown cells (Fig. 4C-D). ELISA assay further revealed that OGD-increased secretion of IL-6, TNF-α and IL-1β were reversed by GPNMB overexpression, while higher levels of these pro-inflammatory cytokines were found in the cell culture media of GPNMB-knockdown cells (Fig. 4E). As shown in Fig. 4F-G, iron accumulation was remarkably enhanced in OGD-treated PC-12 cells. GPNMB overexpression abrogated OGD-induced iron accumulation, while GPNMB knockdown potentiated this effect in PC-12 cells exposed to OGD treatment. Moreover, OGD-mediated changes of total iron, GSH, MDA and ROS were abrogated by GPNMB overexpression. Knockdown of GPNMB exerted opposite effects on these lipid peroxidation markers in PC-12 cell upon OGD treatment (Fig. 4H). In line with the qRT-PCR results, the protein level GPNMB was upregulated by OGD treatment, and OGD-upregulated GPNMB was potentiated or counteracted by GPNMB overexpression or knockdown, respectively. OGD-downregulated GPX4 was attenuated by GPNMB overexpression, while silencing of GPNMB led to a more pronounced reduction of GPX4 in PC-12 cells (Fig. 4I). Together, these findings suggest that GPNMB alleviates OGD-induced impairments in PC-12 cells, possibly via modulating ferroptosis.

### STAT3 acts as a transcriptional activator of GPNMB

**Fig. 5:**
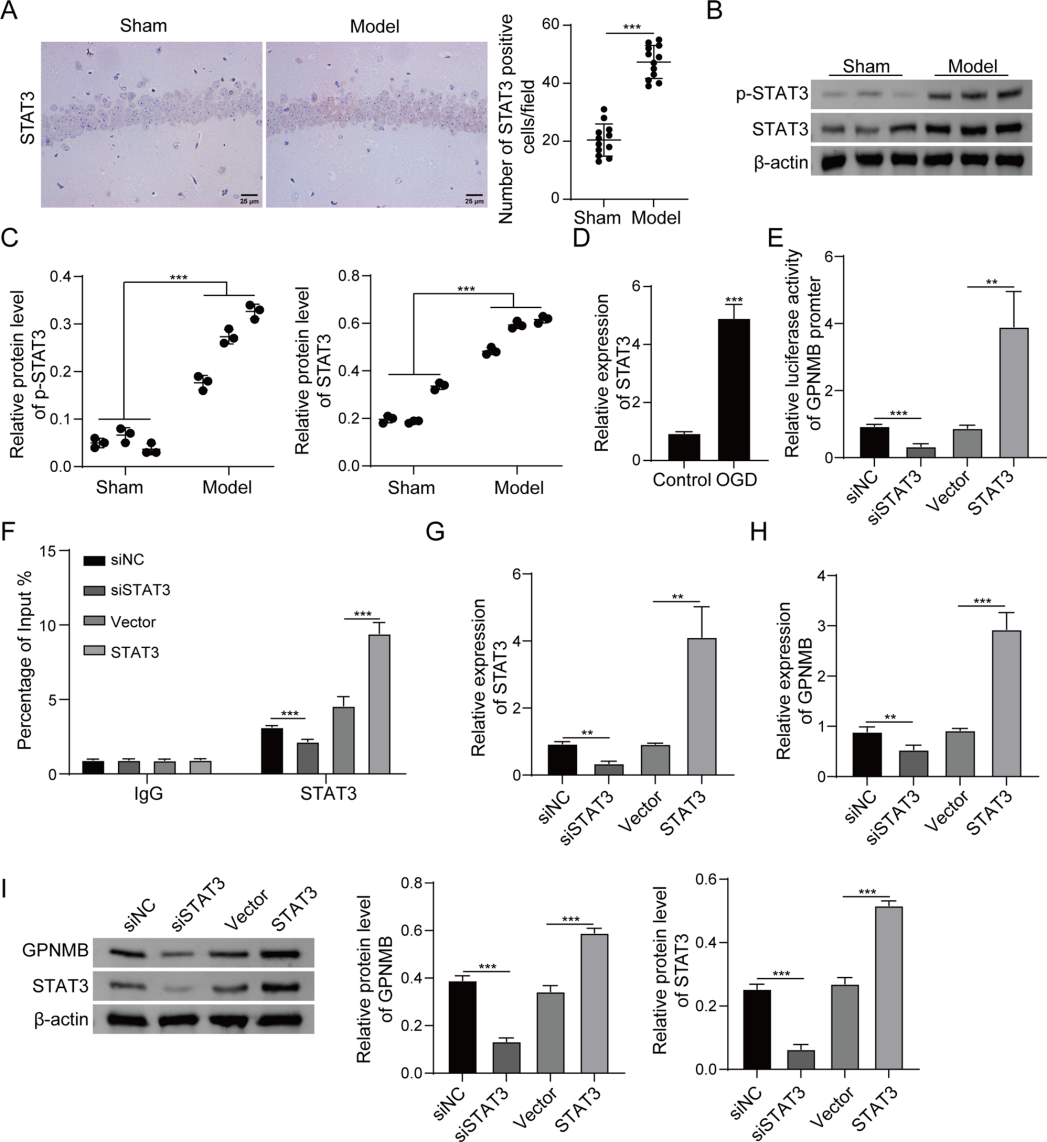
STAT3 acts as a transcriptional activator of GPNMB. (**A**) The immunoreactivity of STAT3 in hippocampus CA1 area was assessed by IHC staining with quantitative analysis. Scale bar, 25 μm. (**B**-**C**) The protein levels of STAT3 and p-STAT3 in hippocampus CA1 area were detected by western blot. *N* = 3. (**D**) The mRNA level of STAT3 in PC-12 cells was detected by qRT-PCR. (**E**) Relative GPNMB promoter activity was measured by dual luciferase reporter assay. (**F**) The direct interaction between STAT3 and GPNMB promoter was detected by ChIP assay. Normal IgG served as a negative control. (**G**-**H**) The mRNA levels of STAT3 and GPNMB in PC-12 cells were detected by qRT-PCR. (**I**) The protein levels of STAT3 and GPNMB in PC-12 cells were detected by western blot. Experiments were performed in triplicate and error bars represent SD of a triplicate set of experiments. Data are shown as mean ± SD. (**A**, **C**-**I**) were analyzed by two tailed Student’s *t*-test. **, *P* < 0.01; ***, *P* < 0.001

Bioinformatics analysis based on Genecard (https://www.genecards.org/, last accessed May 16, 2022) and ConTra software (https://sourceforge.net/projects/contra-cnv/files/CONTRA.V2.0/, last accessed November 16, 2022) predicted the potential STAT3-binding sites in *GPNMB* promoter region. Intriguingly, IHC analysis showed increased STAT3-positive cells in hippocampus CA1 area of model rats (Fig. 5A). In accordance with this finding, western blot confirmed the elevations of STAT3 and p-STAT3 in hippocampal CA1 tissues derived from CO-poisoned rats (Fig. 5B-C). Similar STAT3 upregulation was also found in OGD-treated PC-12 cells (Fig. 5D). The researchers next studied if STAT3 regulated *GPNMB* at transcriptional level. Dual luciferase reporter assay showed that knockdown or overexpression of STAT3 was accompanied with reduced or induced promoter activity of *GPNMB* (Fig. 5E). The direct association between STAT3 and *GPNMB* promoter was also validated by ChIP assay in which antibody against STAT3 successfully enriched *GPNMB* promoter fragment. As expected, lack of STAT3 reduced the enrichment of *GPNMB* promoter fragment, while overexpression of STAT3 exerted an opposite effect (Fig. 5F). Transfection of siSTAT3 or STAT3 overexpression plasmid decreased or increased STAT3 mRNA level in PC-12 cells, respectively (Fig. 5G). qRT-PCR and western blot further showed that STAT3 positively regulated GPNMB expression in PC-12 cells (Fig. 5H-I). These findings illustrate STAT3 activation in hippocampal CA1 tissues of CO-poisoned rats and OGD-treated PC-12 cells, and it positively regulates GPNMB expression at the transcriptional level.

### GPNMB stabilizes HIF-1α by direct binding

**Fig. 6:**
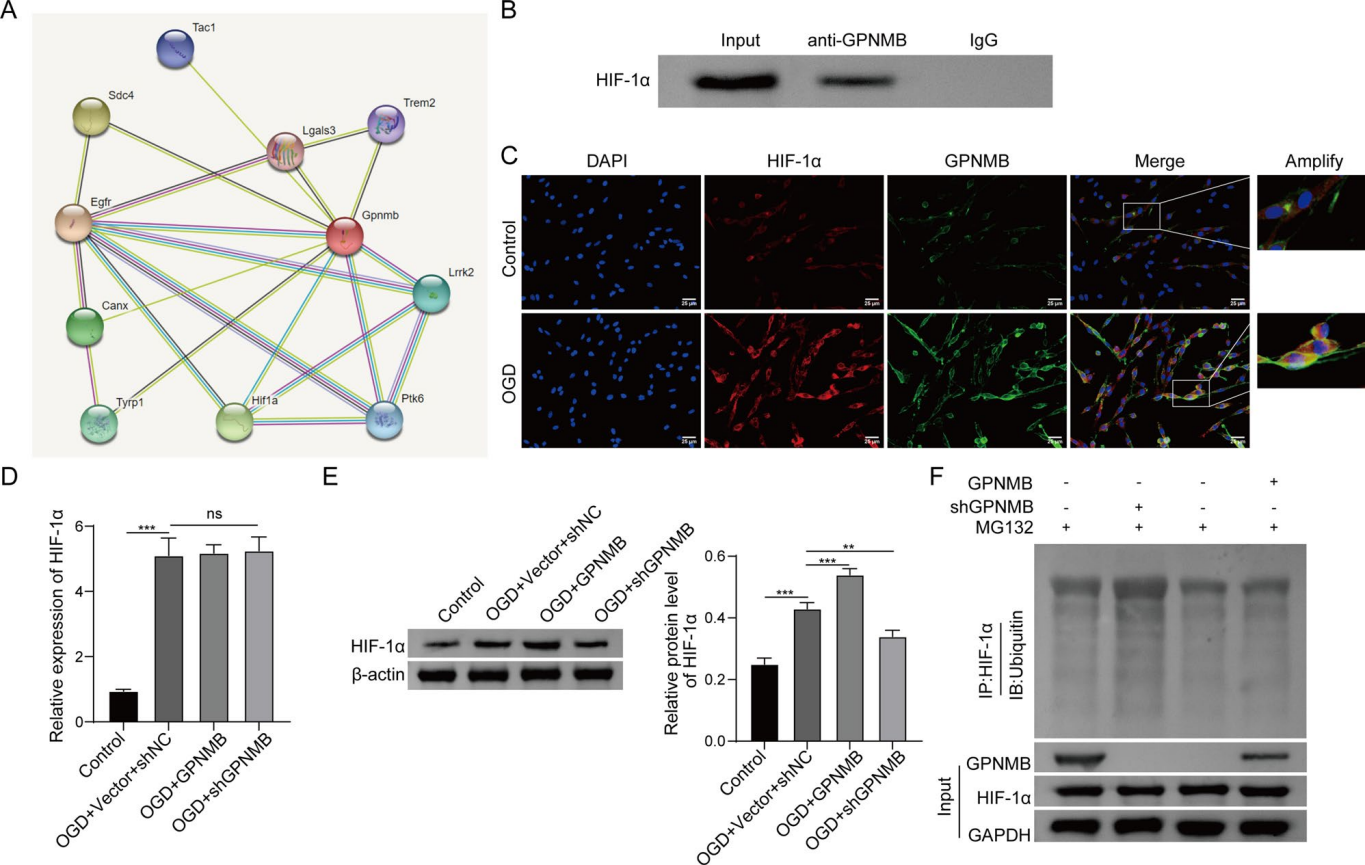
GPNMB stabilizes HIF-1α by direct binding. **A**) Bioinformatics analysis based on STRING database. (**B**) The direct interaction between GPNMB and HIF-1α in OGC-treated PC-12 cells was detected by co-IP. Whole cell lysates and normal IgG served as an input and negative control, respectively. (**C**) The subcellular localizations of GPNMB and HIF-1α in control and OGC-treated PC-12 cells were detected by IF staining. Red, HIF-1α; Green, GPNMB; Blue, DAPI. Scale bar, 50 μm. (**D**) The mRNA level of HIF-1α in PC-12 cells was detected by qRT-PCR. (**E**) The protein level of HIF-1α in PC-12 cells was detected by western blot. (**F**) The ubiquitination of HIF-1α was monitored by co-IP. Whole cell lysates served as an input control. Experiments were performed in triplicate and error bars represent SD of a triplicate set of experiments. Data are shown as mean ± SD. (**D**-**E**) were analyzed by one-way ANOVA followed by *Bonferroni’s multiple comparison tests*. **, *P* < 0.01; ***, *P* < 0.001; ns, not significant

STRING database (https://cn.string-db.org/cgi/input?sessionId=bQFAppYU3tX6&input_page_show_search=on, last accessed April 10, 2023) predicted the putative interaction between GPNMB and HIF-1α (Fig. 6A), which was confirmed by co-IP in OGD-treated PC-12 cells (Fig. 6B). As shown in Fig. 6C, the co-localization of HIF-1α and GPNMB was observed in both control and OGD-treated PC-12 cells, and OGD-treated cells exhibited enhanced signals for both HIF-1α and GPNMB, along with their merged signal. qRT-PCR showed that the mRNA level of HIF-1α was greatly increased in OGD-treated PC-12 cells, while overexpression or knockdown of GPNMB showed no significant effect on HIF-1α mRNA level (Fig. 6D). By contrast, OGD-induced HIF-1α protein expression was potentiated in GPNMB-overexpressing cells, whereas the effect of OGD treatment was abolished by sh-GPNMB (Fig. 6E), indicating that GPNMB might regulate HIF-1α protein level via post-translational modification. Furthermore, co-IP showed that overexpression of GPNMB inhibited the ubiquitination of HIF-1α in the presence of proteasome inhibitor MG132. Silencing of GPNMB facilitated the ubiquitination of HIF-1α in PC-12 cells (Fig. 6F). These data suggest the direct interaction between GPNMB and HIF-1α in PC-12 cells, and GPNMB stabilizes HIF-1α via suppressing its ubiquitin-mediated degradation.

### GPNMB protects against OGD-induced impairments via modulating HIF-1α

**Fig. 7:**
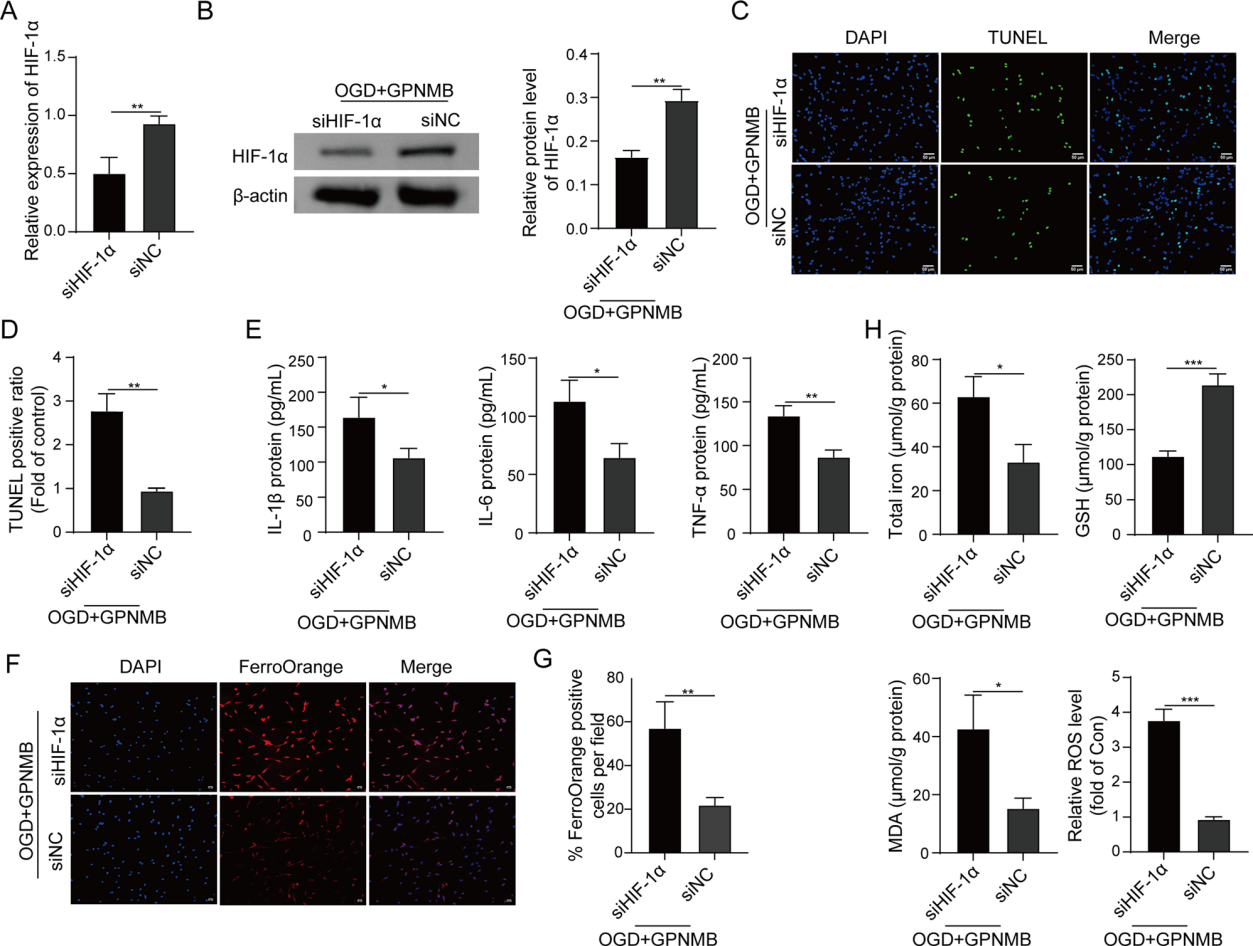
GPNMB protects against OGD-induced impairments via modulating HIF-1α. (**A**) The mRNA level of HIF-1α in PC-12 cells was detected by qRT-PCR. (**B**) The protein level of HIF-1α in OGD-treated GPNMB-overexpressing PC-12 cells was detected by western blot. (**C**-**D**) Cell apoptosis in OGD-treated GPNMB-overexpressing PC-12 cells was assessed by TUNEL assay with quantitative analysis. Green, TUNEL; Blue, DAPI. Scale bar, 50 μm. (**E**) The secretion of pro-inflammatory cytokines in cell culture supernatant were measured by ELISA assay. (**F**-**G**) The levels of iron, GSH, MDA and ROS in OGD-treated GPNMB-overexpressing PC-12 cells were measured using commercial kits. Experiments were performed in triplicate and error bars represent SD of a triplicate set of experiments. Data are shown as mean ± SD. (**A**-**B**, **D**-**E**, **G**-**H**) were analyzed by two tailed Student’s *t*-test. *, *P* < 0.05; **, *P* < 0.01; ***, *P* < 0.001

The role of HIF-1α in GPNMB-regulated ferroptosis was investigated by knockdown experiments in PC-12 cells. As presented in Fig. 7A, successful HIF-1α knockdown in PC-12 cells was confirmed by western blot. In OGD-treated GPNMB-overexpressing PC-12 cells, silencing of HIF-1α successfully decreased HIF-1α protein level as detected by western blot (Fig. 7B). TUNEL assay revealed that the protective effect of GPNMB on OGD-induced apoptosis was reversed by HIF-1α knockdown (Fig. 7C-D). As expected, lack of HIF-1α led to rebounds of IL-6, TNF-α and IL-1β secretion in OGD + GPNMB group, compared with corresponding control (Fig. 7E). In addition, the protective effects of GPNMB on OGD-induced changes of iron, GSH, MDA and ROS were also attenuated by HIF-1α knockdown in PC-12 cells (Fig. 7F-H). Taken together, these data indicate that HIF-1α functions as critical downstream effector in GPNMB-regulated ferroptosis in OGD-treated PC-12 cells.

### Loss or gain of GPNMB takes actions on cognitive impairment, oxidative stress and neuronal ferroptosis of rats with DEACMP

**Fig. 8:**
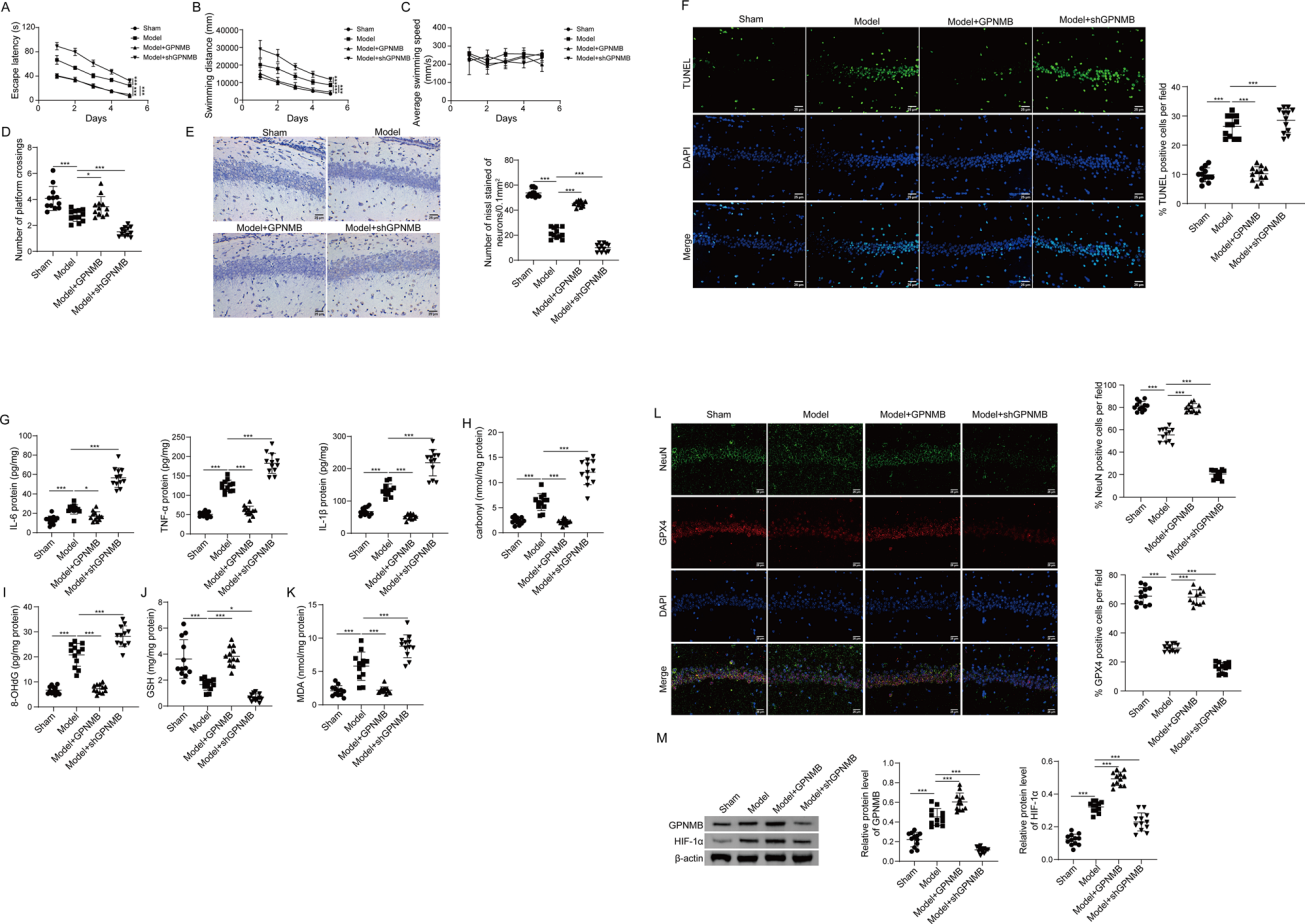
Loss or gain of GPNMB takes actions on cognitive impairment, oxidative stress and neuronal ferroptosis of rats with DEACMP. (**A**) Escape latency was evaluated by MWM tests. (**B**) Swimming distance was evaluated by MWM tests. (**C**) Average swimming swspeed was assessed by MWM tests. (**D**) The number of platform crossing was recorded. For each day of training, data were averaged across five daily trials. (**E**) The morphology of hippocampal neurons was detected by Nissl staining. Scale bar, 25 μm. (**F**) Cell apoptosis in hippocampus CA1 area was assessed by TUNEL assay. Scale bar, 25 μm. (**G**) The levels of pro-inflammatory cytokines in hippocampus CA1 area were measured by ELISA assay. (**H**-**I**) The levels of protein carbonyl content and 8-OHdG in hippocampus CA1 area were assessed using commercial kits. (**J**-**K**) The levels of GSH and MDA in hippocampus CA1 area were measured using commercial kits. (**L**) The immunoreactivities of NeuN and GPX4 in hippocampus CA1 area were detected by IF staining. Green, NeuN; Red, GPX4; Blue, DAPI. Scale bar, 25 μm. (**M**) The protein levels of GPNMB and HIF-1α were detected by western blot. *N* = 12 per group. (**A**-**E**, **G**-**K**, **M**) were analyzed by one-way ANOVA followed by *Bonferroni’s multiple comparison tests*. *, *P* < 0.05; ***, *P* < 0.001

To study the therapeutic potential of GPNMB in vivo, gain- and loss-of-function experiments were conducted in CO-poisoned rats. In accordance with the in vitro findings, MWM tests revealed that overexpression of GPNMB rescued CO poisoning-increased escape latency and swimming distance, as well as CO poisoning-decreased swimming speed and number of platform crossing. By contrast, silencing of GPNMB exacerbated CO poisoning-induced cognitive impairment in model rats (Fig. 8A-D). Nissl staining also revealed that GPNMB overexpression restored CO-induced reduction of Nissl body in hippocampal neurons, while GPNMB knockdown exacerbated this decrease in model rats (Fig. 8E). TUNEL assay showed that CO poisoning-increased apoptotic rate of hippocampal neurons was abolished by GPNMB overexpression, while GPNMB knockdown exerted an opposite effect on neuronal apoptosis (Fig. 8F). Moreover, GPNMB overexpression exhibited a protective effect on inflammation in which the elevation of IL-6, TNF-α and IL-1β in CA1 hippocampal tissues were downregulated in Model + GPNMB group. On the contrary, lack of GPNMB exacerbated CO poisoning-increased inflammation in hippocampus CA1 area as detected by ELISA assay (Fig. 8G). Furthermore, CO poisoning-induced changes of carbonyl content, 8-OHdG, GSH and MDA were also reversed by GPNMB overexpression, but potentiated by GPNMB knockdown in rat CA1 hippocampal tissues (Fig. 8H-K). IF staining revealed that GPNMB overexpression led to rebounds of NeuN-positive and GPX4-positive cells in hippocampus CA1 area, while GPNMB knockdown exerted an opposite effect (Fig. 8L). It is worth noting that the successful upregulation or downregulation of GPNMB in hippocampus CA1 area was confirmed by western blot. HIF-1α was also markedly increased in CO-poisoned rats. A more prominent upregulation of HIF-1α was observed in Model + GPNMB group, while knockdown of GPNMB attenuated CO poisoning-upregulated HIF-1α in hippocampus CA1 area (Fig. 8M). Together, these findings indicate the protective effects of GPNMB on CO poisoning-induced cognitive impairment, inflammation, oxidation and neuronal ferroptosis in vivo.

## Discussion

CO is a colorless and odorless gas, exhibits high affinity for hemoglobin and other heme-containing proteins, such as mitochondrial cytochrome c oxidase (COX) and myoglobin, resulting in neurological deficits and multi-organ failure [3, 35]. Patients with acute CO poisoning benefit from prompt treatment of 100% oxygen, while ~ 50% of patients with severe CO poisoning develop DEACMP [3, 36]. In the present study, the findings demonstrated that upregulated GPNMB protected against CO poisoning-induced impairments via inhibiting neuronal ferroptosis in the in vitro and in vivo models. In addition, STAT3 or HIF-1α was identified as a transcriptional activator or a downstream effector of GPNMB in DEACMP, respectively. These findings provided novel insight into the targeted therapeutic strategy for DEACMP.

Ferroptosis, a nonapoptotic form of RCD, is characterized by iron accumulation and lipid peroxidation, as well as smaller mitochondria with condensed mitochondrial membrane density [37]. Accumulating evidence suggests that the collapse of system xc^−^ and the GPX4 antioxidant enzyme leads to the accumulation of iron-mediated lipid peroxidation, thereby triggering ferroptosis. Besides the classical pathway, recent studies have illustrated that GPX4-independent signaling also contributes to ferroptosis. For instance, ferroptosis suppressor protein 1 (FSP1) catalyzes the regeneration of the reduced form of coenzyme Q_10_ to suppress ferroptosis [38, 39]. In recent years, a number of studies have supported the crucial role of ferroptosis in neurological diseases, including ischemic stroke, Alzheimer’s disease, PD and other neurodegenerative disorders [12, 14, 40]. For instance, it has been reported that HIF-1α ameliorates cerebral ischemic injury by modulating ferroptosis through the FPN1/Nrf2/HO-1 axis [14]. However, the role of ferroptosis in DEACMP remains undefined. This study found that Fer-1 abrogated CO poisoning-induced cognitive impairment, as well as inflammation and oxidation in the hippocampal CA1 area of DEACMP rats. Additionally, OGD treatment also increased the levels of iron, MDA and ROS, along with the decreased expression of GPX4 in PC-12 cells. An earlier study has illustrated that CO exposure-induced delayed amnesia may be attributed to delayed neuronal death in the hippocampal CA1 subfield [41]. Subsequent reports have also unraveled the pathogenesis of delayed neuronal death in the CA1 region, such as lipid peroxidation, inflammation, mitochondrial dysfunction and adaptive immunological responses [42–44]. Therefore, researchers have mainly focused on the changes of hippocampal CA1 region in DEACMP [45, 46]and the histological changes and ferroptosis in the CA1 region was also investigated in this study. Ferroptosis and GPNMB expression in different regions of the hippocampus merit in-depth investigation in the furture study. The in vivo and in vitro findings indicate the important roles of ferroptosis in DEACMP, suggesting that pharmacological targeting of ferroptosis by a GPX4 activator or FSP1 might be a promising treatment to protect against CO poisoning-induced neurological impairments. Besides ferroptosis, previous researches have also demonstrated the neuroprotective roles of the Nrf2/GCLC and GSK-3β/Tau pathways in acute carbon monoxide poisoning [24, 26]. The crosstalk between ferroptosis and other signalings should be explored in the future research.

GPNMB is widely expressed in multiple tissues and exhibits different functions in each tissue. For instance, GPNMB is implicated in osteoblast and osteoclast differentiation, degeneration /regeneration of skeletal muscle extracellular matrix and T cell activation [47–50]. Given its ubiquitous expression in the central nervous system (CNS), the functions of GPNMB have gained increasing attention recently. It has been reported that the extracellular fragment (ECF) of GPNMB improves memory in mice, which is accompanied by the increased hippocampal GluA1 expression [51]. Subsequent study has illustrated that GPNMB ECF exerts neuroprotective effects through activating PI3K/Akt and MAPK/ERK signalings via Na^+^/K^+^-ATPase [52]. In accordance with the results of RNA sequencing and clinical data, these findings showed the elevated expression of GPNMB in hippocampal CA1 area and OGD-treated PC-12 cells. GPNMB is a membrane-bound protein, however, previous studies and bioinformatics analysis have also illustrated that GPNMB is expressed in the plasma membrane, as well as in the cytoplasm [19, 53, 54]. This might be attributed to the localization of different isoforms of GPNMB. The precise cellular distribution of GPNMB in the hippocampus requires further investigation in the future study. Gain- and loss-of function experiments also showed the neuroprotective effects of GPNMB in vitro and in vivo. If the ECF of GPNMB acted as a pivotal player in DEACMP merits further investigation in future study. In addition, previous studies have demonstrated the anti-oxidative role of GPNMB in hepatic macrophages and stellate cells [55]as well as the involvement of GPNMB in the regulation of lipogenesis in white adipose tissue [22]. Lipid metabolism and oxidation are involved in ferroptosis [56]. Consistently, the present study reported the anti-ferroptotic function of GPNMB in vitro and in vivo in which overexpression of GPNMB decreased the oxidative stress markers and iron levels, but increased GSH and GPX4 levels, indicating that GPNMB suppresses ferroptosis in DEACMP.

Bioinformatics analysis based on GeneCard predicted the potential transcription factors of *GPNMB*. Among the putative transcription factors, the well-known transcription factor and intracellular transducer STAT3 attracted attention due to its critical role in the CNS. More importantly, STAT3 is a transcription factor for Manganese superoxide dismutase (Mn-SOD), and it is involved in neuroprotection against cerebral ischemia [57]. Moreover, the JAK2/STAT3 signaling enhances recovery of neurological function after ischemic stroke [58, 59]. In line with previous findings, the induction of STAT3 was observed in hippocampal CA1 area of CO-poisoned rats and OGD-treated PC-12 cells, and it functioned as a transcription activator of GPNMB. These findings suggest that STAT3 was activated upon CO poisoning, thus providing neuroprotection in DEACMP through upregulating GPNMB.

Besides the upstream regulator STAT3, HIF-1α was identified as a direct binding partner and downstream effector of GPNMB in DEACMP. The role of HIF-1α in neurological disorders remains controversial. A previous study has illustrated that ischemic preconditioning upregulates HIF-1α level in hippocampal CA1 area, thus inducing VEGF expression and activating NF-κB signaling to protect against cerebral ischemia [60]. The ROS/HIF-1α/β-catenin signaling contributes to delayed hyperbaric oxygen (HBO)-facilitated neurogenesis and neurofunctional recovery after stroke [61]. On the contrary, a recent study has reported that the STAT3/HIF-1α/PTRF axis exacerbates cerebral ischemia/reperfusion (I/R) injury [62]. The findings demonstrated the neuroprotective role of HIF-1α in DEACMP. GPNMB co-localized with HIF-1α and maintained its stability. Functional experiments showed that lack of HIF-1α abrogated the neuroprotective effects of GPNMB on inflammation, oxidative stress and ferroptosis in vitro, and the neuroprotection of GPNMB was also accompanied by the induction of HIF-1α in vivo, indicating the function of HIF-1α in GPNMB-regulated ferroptosis. Intriguingly, HIF-1α induces the expression of ACSL4, a ferroptosis mediator, in ischemic stroke [63]. Sorafenib protects against liver injury via triggering hepatic stellate cell (HSC) ferroptosis through HIF-1α/SLC7A11 signaling [64]. In addition to these findings, these data broaden the understanding of HIF-1α-regulated ferroptosis in DEACMP. It is worth noting that GPNMB binds to Na^+^/K^+^-ATPase via the extracellular domain [52]and this study demonstrated the direct interaction between GPNMB and HIF-1α, raising the possibility that the intracellular domain of GPNMB might be responsible for binding with HIF-1α. Further study with GPNMB truncation is required to test this hypothesis. Moreover, the expression of GPNMB and HIF-1α are expected to change over time in DEACMP, while the present study focused on certain time-point in vivo and in vitro. A dose- or time-dependent study is needed to investigate the physiological changes of GPNMB and HIF-1α in DEACMP.

Despite the current findings, this study has some limitations. First, PC-12 cells originate from the adrenal medulla, but these cells have been extensively used in neuroscience research, including studies on neurosecretion, neurotoxicity and neuroprotection. In particular, the OGD-treated PC-12 cell model has been used to study DEACMP pathogenesis [23, 65]. Even though NGF-induced differentiated PC-12 cells were used in this study, further exploration and validation using primary hippocampal CA1 neuronal cells should be a key point in our future study. Second, in vivo injection faces limitations in achieving 100% transfection efficiency. Future investigation may consider employing a transgenic animal model to further validate the current findings. Third, the lack of sham-group validation for the in vivo genetic manipulations should be addressed in future study.

In conclusion, this study reported that the STAT3/GPNMB/HIF-1α axis protects against DEACMP by inhibiting neuronal ferroptosis.

## Electronic supplementary material

Below is the link to the electronic supplementary material.

Supplementary Material 1

## Data Availability

All data generated or analyzed are included in this article. Further inquiries can be directed to the corresponding author.

## Abbreviations

ACMP: Acute carbon monoxide poisoning
ALS: Amyotrophic lateral sclerosis
ChIP: Chromatin immunohistochemistry
CNS: central nervous system
CO: carbon monoxide
co-IP: Co-immunoprecipitation
COX: Cytochrome c oxidase
DEACMP: Delayed encephalopathy after acute carbon monoxide poisoning
ECF: Extracellular fragment
Fer-1: Ferrostain-1
FSP1: Ferroptosis suppressor protein 1
GPNMB: Glycoprotein non-metastatic melanoma protein B
GPX4: Glutathione peroxidase 4
HBO: Hyperbaric oxygen
H&E: Hematoxylin and eosin
HSC: Hepatic stellate cell
ICV: Intracerebroventricular
IF: Immunofluorescence
IHC: Immunohistochemistry
I/R: Ischemia/reperfusion
Mn-SOD: Manganese superoxide dismutase
MWM: Morris Water Maze
NGF: Nerve growth factor
PD: Parkinson’s disease
RCD: Regulated cell death
ROS: Reactive oxygen species
SYP: Synaptophysin
OGD: Oxygen-glucose deprivation
PD: Parkinson’s disease
PFA: Paraformaldehyde

## References

[1] Mattiuzzi C, Lippi G (2020) Worldwide epidemiology of carbon monoxide poisoning. Hum Exp Toxicol 39:387–392 31789062 10.1177/0960327119891214

[2] Cui P, Jin Y, Feng H, Li Z, Ding S, Li Y (2022) Burden of carbon monoxide poisoning in china, 1990–2019: A systematic analysis of data from the global burden of disease study 2019. Front Public Health 10:930784 35968482 10.3389/fpubh.2022.930784 PMC9371476

[3] Rose JJ, Wang L, Xu Q, McTiernan CF, Shiva S, Tejero J, Gladwin MT (2017) Carbon monoxide poisoning: pathogenesis, management, and future directions of therapy. Am J Respir Crit Care Med 195:596–606 27753502 10.1164/rccm.201606-1275CI PMC5363978

[4] Xu XM, Luo H, Rong BB, Zheng XM, Wang FT, Zhang SJ, Li ZX (2019) Management of delayed encephalopathy after CO poisoning: an evidence-based narrative review. Med (Baltim) 98:e18199 10.1097/MD.0000000000018199 PMC6919536 31804341

[5] Jiang X, Stockwell BR, Conrad M (2021) Ferroptosis: mechanisms, biology and role in disease. Nat Rev Mol Cell Biol 22:266–282 33495651 10.1038/s41580-020-00324-8 PMC8142022

[6] Huang YQ, Peng ZR, Huang FL, Yang AL (2020) Mechanism of delayed encephalopathy after acute carbon monoxide poisoning. Neural Regen Res 15:2286–2295 32594050 10.4103/1673-5374.284995 PMC7749483

[7] Wang S, Xiong B, Tian Y, Hu Q, Jiang X, Zhang J, Chen L, Wang R, Li M, Zhou X, Zhang T, Ge H, Yu A (2024) Targeting ferroptosis promotes functional recovery by mitigating white matter injury following acute carbon monoxide poisoning. Mol Neurobiol 61:1157–1174 37697220 10.1007/s12035-023-03603-5

[8] Wang Y, Zhou Z, Zhang D, Jiang Y (2025) Predictors of delayed encephalopathy after acute carbon monoxide poisoning: a literature review. Front Med (Lausanne) 12:1559264 40206479 10.3389/fmed.2025.1559264 PMC11979149

[9] Akyol S, Erdogan S, Idiz N, Celik S, Kaya M, Ucar F, Dane S, Akyol O (2014) The role of reactive oxygen species and oxidative stress in carbon monoxide toxicity: an in-depth analysis. Redox Rep 19:180–189 24773392 10.1179/1351000214Y.0000000094 PMC6837723

[10] Ye Q, Zeng C, Dong L, Wu Y, Huang Q, Wu Y (2019) Inhibition of ferroptosis processes ameliorates cognitive impairment in Kainic acid-induced Temporal lobe epilepsy in rats. Am J Transl Res 11:875–884 30899387 PMC6413264

[11] Ye Q, Zeng C, Luo C, Wu Y (2020) Ferrostatin-1 mitigates cognitive impairment of epileptic rats by inhibiting P38 MAPK activation. Epilepsy Behav 103:106670 31864943 10.1016/j.yebeh.2019.106670

[12] Ou M, Jiang Y, Ji Y, Zhou Q, Du Z, Zhu H, Zhou Z (2022) Role and mechanism of ferroptosis in neurological diseases. Mol Metab 61:101502 35447365 10.1016/j.molmet.2022.101502 PMC9170779

[13] An S, Shi J, Huang J, Li Z, Feng M, Cao G (2024) HIF-1alpha induced by hypoxia promotes peripheral nerve injury recovery through regulating ferroptosis in DRG neuron. Mol Neurobiol 61:6300–6311 38291291 10.1007/s12035-024-03964-5

[14] Yao H, Tian J, Cheng S, Dou H, Zhu Y (2024) The mechanism of hypoxia-inducible factor-1alpha enhancing the transcriptional activity of transferrin Ferroportin 1 and regulating the Nrf2/HO-1 pathway in ferroptosis after cerebral ischemic injury. Neuroscience 559:26–38 39168172 10.1016/j.neuroscience.2024.08.025

[15] Taya M, Hammes SR (2018) Glycoprotein Non-Metastatic Melanoma Protein B (GPNMB) and Cancer: A Novel Potential Therapeutic Target. Steroids 133:102-7 10.1016/j.steroids.2017.10.013 PMC6166407 29097143

[16] Huang JJ, Ma WJ, Yokoyama S (2012) Expression and immunolocalization of gpnmb, a glioma-associated glycoprotein, in normal and inflamed central nervous systems of adult rats. Brain Behav 2:85–96 22574278 10.1002/brb3.39 PMC3345354

[17] Tanaka H, Shimazawa M, Kimura M, Takata M, Tsuruma K, Yamada M, Takahashi H, Hozumi I, Niwa J, Iguchi Y, Nikawa T, Sobue G, Inuzuka T, Hara H (2012) The potential of GPNMB as novel neuroprotective factor in amyotrophic lateral sclerosis. Sci Rep 2:573 22891158 10.1038/srep00573 PMC3417778

[18] Nakano Y, Suzuki Y, Takagi T, Kitashoji A, Ono Y, Tsuruma K, Yoshimura S, Shimazawa M, Iwama T, Hara H (2014) Glycoprotein nonmetastatic melanoma protein B (GPNMB) as a novel neuroprotective factor in cerebral ischemia-reperfusion injury. Neuroscience 277:123–131 25010402 10.1016/j.neuroscience.2014.06.065

[19] Neal ML, Boyle AM, Budge KM, Safadi FF, Richardson JR (2018) The glycoprotein GPNMB attenuates astrocyte inflammatory responses through the CD44 receptor. J Neuroinflammation 15:73 29519253 10.1186/s12974-018-1100-1 PMC5842560

[20] Deng L, He S, Guo N, Tian W, Zhang W, Luo L (2023) Molecular mechanisms of ferroptosis and relevance to inflammation. Inflamm Res 72:281–299 36536250 10.1007/s00011-022-01672-1 PMC9762665

[21] Sun Y, Chen P, Zhai B, Zhang M, Xiang Y, Fang J, Xu S, Gao Y, Chen X, Sui X, Li G (2020) The emerging role of ferroptosis in inflammation. Biomed Pharmacother 127:110108 32234642 10.1016/j.biopha.2020.110108

[22] Gong XM, Li YF, Luo J, Wang JQ, Wei J, Wang JQ, Xiao T, Xie C, Hong J, Ning G, Shi XJ, Li BL, Qi W, Song BL (2019) Gpnmb secreted from liver promotes lipogenesis in white adipose tissue and aggravates obesity and insulin resistance. Nat Metab 1:570–583 32694855 10.1038/s42255-019-0065-4

[23] Liu ZL, Bian M, Pang L (2022) LncRNA CRNDE deteriorates delayed encephalopathy after acute carbon monoxide poisoning to inactivate AKT/GSK3beta/beta-catenin pathway via miR-212-5p. Neurotox Res 40:1208–1222 35852716 10.1007/s12640-022-00518-2

[24] Zhou XD, Wang JL, Guo DD, Jiang WW, Li ZK, Wang L, Zou Y, Bi MJ, Li Q (2021) Neuroprotective effect of targeted regulatory Nrf2 gene on rats with acute brain injury induced by carbon monoxide poisoning. Environ Toxicol 36:1742–1757 34032369 10.1002/tox.23295

[25] Lopachev A, Volnova A, Evdokimenko A, Abaimov D, Timoshina Y, Kazanskaya R, Lopacheva O, Deal A, Budygin E, Fedorova T, Gainetdinov R (2019) Intracerebroventricular injection of Ouabain causes mania-like behavior in mice through D2 receptor activation. Sci Rep 9:15627 31666560 10.1038/s41598-019-52058-z PMC6821712

[26] Su C, Zhao N, Zou J, Yan X (2020) TDZD-8 alleviates delayed neurological sequelae following acute carbon monoxide poisoning involving Tau protein phosphorylation. Inhal Toxicol 32:79–85 32188325 10.1080/08958378.2020.1741739

[27] Bao L, Zhao J, Gregersen H (2024) Association between jejunal remodeling in fasting rats and hypersensitivity of intestinal afferent nerves to mechanical stimulation. Biomech Model Mechanobiol 23:73–86 37548873 10.1007/s10237-023-01758-7

[28] Deng Q, Gao Y, Wang Y, Mao J, Yan Y, Yang Z, Cong Y, Yang Y, Wan S (2024) LSD1 Inhibition by Tranylcypromine hydrochloride reduces alkali burn-induced corneal neovascularization and ferroptosis by suppressing HIF-1alpha pathway. Front Pharmacol 15:1411513 39130627 10.3389/fphar.2024.1411513 PMC11316257

[29] Stroh MA, Winter MK, Swerdlow RH, McCarson KE, Zhu H (2016) Loss of NCB5OR in the cerebellum disturbs iron pathways, potentiates behavioral abnormalities, and exacerbates harmaline-induced tremor in mice. Metab Brain Dis 31:951–964 27188291 10.1007/s11011-016-9834-x PMC5929129

[30] Schwark R, Andrade R, Bykhovskaia M (2022) Synapsin II directly suppresses epileptic seizures in vivo. Brain Sci 12:325 10.3390/brainsci12030325 PMC8946686 35326282

[31] Sierra-Fonseca JA, Najera O, Martinez-Jurado J, Walker EM, Varela-Ramirez A, Khan AM, Miranda M, Lamango NS, Roychowdhury S (2014) Nerve growth factor induces neurite outgrowth of PC12 cells by promoting Gbetagamma-microtubule interaction. BMC Neurosci 15:132 25552352 10.1186/s12868-014-0132-4 PMC4302597

[32] Yuan J, Zeng L, Sun Y, Wang N, Sun Q, Cheng Z, Wang Y (2018) SH2B1 protects against OGD/R–induced apoptosis in PC12 cells via activation of the JAK2/STAT3 signaling pathway. Mol Med Rep 18:2613–2620 30015896 10.3892/mmr.2018.9265 PMC6102733

[33] Barnhart CD, Yang D, Lein PJ (2015) Using the Morris water maze to assess Spatial learning and memory in weanling mice. PLoS ONE 10:e0124521 25886563 10.1371/journal.pone.0124521 PMC4401674

[34] Liu Y, Feng H, Fu H, Wu Y, Nie B, Wang T (2022) Altered functional connectivity and topology structures in default mode network induced by inflammatory exposure in aged rat: A resting-state functional magnetic resonance imaging study. Front Aging Neurosci 14:1013478 36466609 10.3389/fnagi.2022.1013478 PMC9714678

[35] Kinoshita H, Turkan H, Vucinic S, Naqvi S, Bedair R, Rezaee R, Tsatsakis A (2020) Carbon monoxide poisoning. Toxicol Rep 7:169–173 32015960 10.1016/j.toxrep.2020.01.005 PMC6992844

[36] Han ST, Bhopale VM, Thom SR (2007) Xanthine oxidoreductase and neurological sequelae of carbon monoxide poisoning. Toxicol Lett 170:111–115 17433579 10.1016/j.toxlet.2007.02.006 PMC2723845

[37] Chen X, Yu C, Kang R, Tang D (2020) Iron metabolism in ferroptosis. Front Cell Dev Biol 8:590226 33117818 10.3389/fcell.2020.590226 PMC7575751

[38] Bersuker K, Hendricks JM, Li Z, Magtanong L, Ford B, Tang PH, Roberts MA, Tong B, Maimone TJ, Zoncu R, Bassik MC, Nomura DK, Dixon SJ, Olzmann JA (2019) The CoQ oxidoreductase FSP1 acts parallel to GPX4 to inhibit ferroptosis. Nature 575:688–692 31634900 10.1038/s41586-019-1705-2 PMC6883167

[39] Doll S, Freitas FP, Shah R, Aldrovandi M, da Silva MC, Ingold I, Goya Grocin A, Xavier da Silva TN, Panzilius E, Scheel CH, Mourao A, Buday K, Sato M, Wanninger J, Vignane T, Mohana V, Rehberg M, Flatley A, Schepers A, Kurz A, White D, Sauer M, Sattler M, Tate EW, Schmitz W, Schulze A, O’Donnell V, Proneth B, Popowicz GM, Pratt DA, Angeli JPF, Conrad M (2019) FSP1 is a glutathione-independent ferroptosis suppressor. Nature 575:693–698 31634899 10.1038/s41586-019-1707-0

[40] Yao MY, Liu T, Zhang L, Wang MJ, Yang Y, Gao J (2021) Role of ferroptosis in neurological diseases. Neurosci Lett 747:135614 33485988 10.1016/j.neulet.2020.135614

[41] Nabeshima T, Katoh A, Ishimaru H, Yoneda Y, Ogita K, Murase K, Ohtsuka H, Inari K, Fukuta T, Kameyama T (1991) Carbon monoxide-induced delayed amnesia, delayed neuronal death and change in acetylcholine concentration in mice. J Pharmacol Exp Ther 256:378–384 1671097

[42] Thom SR (1990) Carbon monoxide-mediated brain lipid peroxidation in the rat. J Appl Physiol (1985) 68:997–1003 10.1152/jappl.1990.68.3.997 2341364

[43] Thom SR, Bhopale VM, Fisher D, Zhang J, Gimotty P (2004) Delayed neuropathology after carbon monoxide poisoning is immune-mediated. Proc Natl Acad Sci U S A 101:13660–13665 15342916 10.1073/pnas.0405642101 PMC518809

[44] Atalay H, Aybek H, Koseoglu M, Demir S, Erbay H, Bolaman AZ, Avci A (2006) The effects of amifostine and dexamethasone on brain tissue lipid peroxidation during oxygen treatment of carbon monoxide-poisoned rats. Adv Ther 23:332–341 16751165 10.1007/BF02850138

[45] Guan L, Zhang YL, Li ZY, Zhu MX, Yao WJ, Zhao JY (2015) Salvianolic acids attenuate rat hippocampal injury after acute CO poisoning by improving blood flow properties. Biomed Res Int 2015:526483 10.1155/2015/526483 PMC4331406 25705671

[46] Zhao N, Liang P, Zhuo X, Su C, Zong X, Guo B, Han D, Yan X, Hu S, Zhang Q, Tie X (2018) After treatment with methylene blue is effective against delayed encephalopathy after acute carbon monoxide poisoning. Basic Clin Pharmacol Toxicol 122:470–480 29151273 10.1111/bcpt.12940

[47] Ogawa T, Nikawa T, Furochi H, Kosyoji M, Hirasaka K, Suzue N, Sairyo K, Nakano S, Yamaoka T, Itakura M, Kishi K, Yasui N (2005) Osteoactivin upregulates expression of MMP-3 and MMP-9 in fibroblasts infiltrated into denervated skeletal muscle in mice. Am J Physiol Cell Physiol 289:C697–707 16100390 10.1152/ajpcell.00565.2004

[48] Abdelmagid SM, Barbe MF, Rico MC, Salihoglu S, Arango-Hisijara I, Selim AH, Anderson MG, Owen TA, Popoff SN, Safadi FF (2008) Osteoactivin, an anabolic factor that regulates osteoblast differentiation and function. Exp Cell Res 314:2334–2351 18555216 10.1016/j.yexcr.2008.02.006

[49] Ripoll VM, Meadows NA, Raggatt LJ, Chang MK, Pettit AR, Cassady AI, Hume DA (2008) Microphthalmia transcription factor regulates the expression of the novel osteoclast factor GPNMB. Gene 413:32–41 18313864 10.1016/j.gene.2008.01.014

[50] Chung JS, Bonkobara M, Tomihari M, Cruz PD Jr., Ariizumi K (2009) The DC-HIL/syndecan-4 pathway inhibits human allogeneic T-cell responses. Eur J Immunol 39:965–974 19350579 10.1002/eji.200838990 PMC2766302

[51] Murata K, Yoshino Y, Tsuruma K, Moriguchi S, Oyagi A, Tanaka H, Ishisaka M, Shimazawa M, Fukunaga K, Hara H (2015) The extracellular fragment of GPNMB (Glycoprotein nonmelanosoma protein B, osteoactivin) improves memory and increases hippocampal GluA1 levels in mice. J Neurochem 132:583–594 25545823 10.1111/jnc.13010

[52] Ono Y, Tsuruma K, Takata M, Shimazawa M, Hara H (2016) Glycoprotein nonmetastatic melanoma protein B extracellular fragment shows neuroprotective effects and activates the PI3K/Akt and MEK/ERK pathways via the Na+/K+-ATPase. Sci Rep 6:23241 26988030 10.1038/srep23241 PMC4796790

[53] Zhu Z, Liu Y, Li X, Zhang L, Liu H, Cui Y, Wang Y, Zhao D (2022) GPNMB mitigates alzheimer’s disease and enhances autophagy via suppressing the mTOR signal. Neurosci Lett 767:136300 34695452 10.1016/j.neulet.2021.136300

[54] Li T, Zhang Y, Lu Q, Lei L, Du J, Lu X (2023) GPNMB ameliorates neuroinflammation via the modulation of ampk/nfkappab signaling pathway after SAH in mice. J Neuroimmune Pharmacol 18:628–639 37919457 10.1007/s11481-023-10087-6 PMC10769934

[55] Katayama A, Nakatsuka A, Eguchi J, Murakami K, Teshigawara S, Kanzaki M, Nunoue T, Hida K, Wada N, Yasunaka T, Ikeda F, Takaki A, Yamamoto K, Kiyonari H, Makino H, Wada J (2015) Beneficial impact of Gpnmb and its significance as a biomarker in nonalcoholic steatohepatitis. Sci Rep 5:16920 26581806 10.1038/srep16920 PMC4652285

[56] Dixon SJ, Lemberg KM, Lamprecht MR, Skouta R, Zaitsev EM, Gleason CE, Patel DN, Bauer AJ, Cantley AM, Yang WS, Morrison B (2012) 3rd, Stockwell BR: ferroptosis: an iron-dependent form of nonapoptotic cell death. Cell 149:1060–1072 22632970 10.1016/j.cell.2012.03.042 PMC3367386

[57] Jung JE, Kim GS, Narasimhan P, Song YS, Chan PH (2009) Regulation of Mn-superoxide dismutase activity and neuroprotection by STAT3 in mice after cerebral ischemia. J Neurosci 29:7003–7014 19474327 10.1523/JNEUROSCI.1110-09.2009 PMC2712132

[58] Hu GQ, Du X, Li YJ, Gao XQ, Chen BQ, Yu L (2017) Inhibition of cerebral ischemia/reperfusion injury-induced apoptosis: Nicotiflorin and JAK2/STAT3 pathway. Neural Regen Res 12:96–102 28250754 10.4103/1673-5374.198992 PMC5319249

[59] Raible DJ, Frey LC, Brooks-Kayal AR (2014) Effects of JAK2-STAT3 signaling after cerebral insults. JAKSTAT 3:e29510 25105066 10.4161/jkst.29510 PMC4124058

[60] Lee JC, Tae HJ, Kim IH, Cho JH, Lee TK, Park JH, Ahn JH, Choi SY, Bai HC, Shin BN, Cho GS, Kim DW, Kang IJ, Kwon YG, Kim YM, Won MH, Bae EJ (2017) Roles of HIF-1alpha, VEGF, and NF-kappaB in ischemic Preconditioning-Mediated neuroprotection of hippocampal CA1 pyramidal neurons against a subsequent transient cerebral ischemia. Mol Neurobiol 54:6984–6998 27785755 10.1007/s12035-016-0219-2

[61] Hu Q, Liang X, Chen D, Chen Y, Doycheva D, Tang J, Tang J, Zhang JH (2014) Delayed hyperbaric oxygen therapy promotes neurogenesis through reactive oxygen species/hypoxia-inducible factor-1alpha/beta-catenin pathway in middle cerebral artery occlusion rats. Stroke 45:1807–1814 24757104 10.1161/STROKEAHA.114.005116 PMC4102647

[62] Jin W, Zhao J, Yang E, Wang Y, Wang Q, Wu Y, Tong F, Tan Y, Zhou J, Kang C (2022) Neuronal STAT3/HIF-1alpha/PTRF axis-mediated bioenergetic disturbance exacerbates cerebral ischemia-reperfusion injury via PLA2G4A. Theranostics 12:3196–3216 35547748 10.7150/thno.71029 PMC9065197

[63] Cui Y, Zhang Y, Zhao X, Shao L, Liu G, Sun C, Xu R, Zhang Z (2021) ACSL4 exacerbates ischemic stroke by promoting ferroptosis-induced brain injury and neuroinflammation. Brain Behav Immun 93:312–321 33444733 10.1016/j.bbi.2021.01.003

[64] Yuan S, Wei C, Liu G, Zhang L, Li J, Li L, Cai S, Fang L (2022) Sorafenib attenuates liver fibrosis by triggering hepatic stellate cell ferroptosis via HIF-1alpha/SLC7A11 pathway. Cell Prolif 55:e13158 34811833 10.1111/cpr.13158 PMC8780895

[65] Liu Z, Bian M, Pang L (2023) LncRNA CRNDE binds hnRNPA1 to facilitate carbon monoxide poisoning-induced delayed encephalopathy via inhibiting UCHL5-mediated SMO deubiquitination. Metab Brain Dis 38:1097–1113 36648699 10.1007/s11011-022-01157-4

