# Correction: Reciprocal regulation of GPNMB/HIF-1α for inhibition of neuronal ferroptosis in delayed encephalopathy after acute carbon monoxide poisoning

**DOI:** 10.1186/s40478-025-02088-8

**Published:** 2025-08-13

**Authors:** Zuolong Liu, Lanyue Sun, Nan Gao, Wei Li, Li Pang

**Affiliations:** 1 Department of Emergency, The First Hospital of Jilin University, No. 1 Xinmin Road, Changchun, 130021 Jilin Province P.R. China; 2 Medical Quality Control Office, The Third Affiliated Hospital of Changchun, University of Chinese Medicine, Changchun, 130118 Jilin Province P.R. China

**Correction: Acta Neuropathologica Communications (2025) 13:154**

10.1186/s40478-025-02069-x

In this article [1], Chart 1 appeared incorrectly and have now been corrected in the original publication. For completeness and transparency, the old incorrect versions are displayed below.

Incorrect Chart 1

**Chart. 1:**
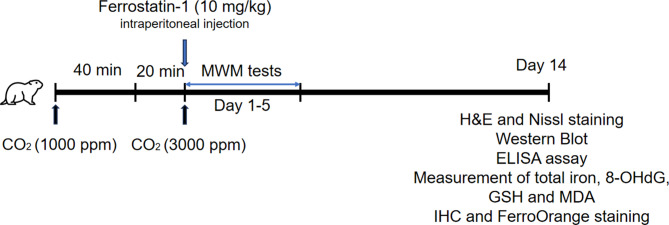
A timeline diagram for summarizing animal experimental design

Correct Chart 1

**Chart. 1:**
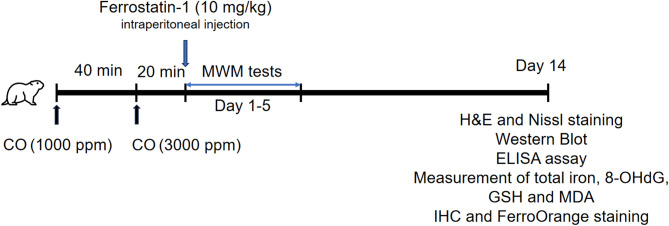
A timeline diagram for summarizing animal experimental design
