# Supplementary figures and images for: Reciprocal regulation of GPNMB/HIF-1α for Inhibition of neuronal ferroptosis in delayed encephalopathy after acute carbon monoxide poisoning
Source: Acta Neuropathol Commun. 2025 Jul 14;13:154. doi: 10.1186/s40478-025-02069-x (PMC12257678; doi:10.1186/s40478-025-02069-x)

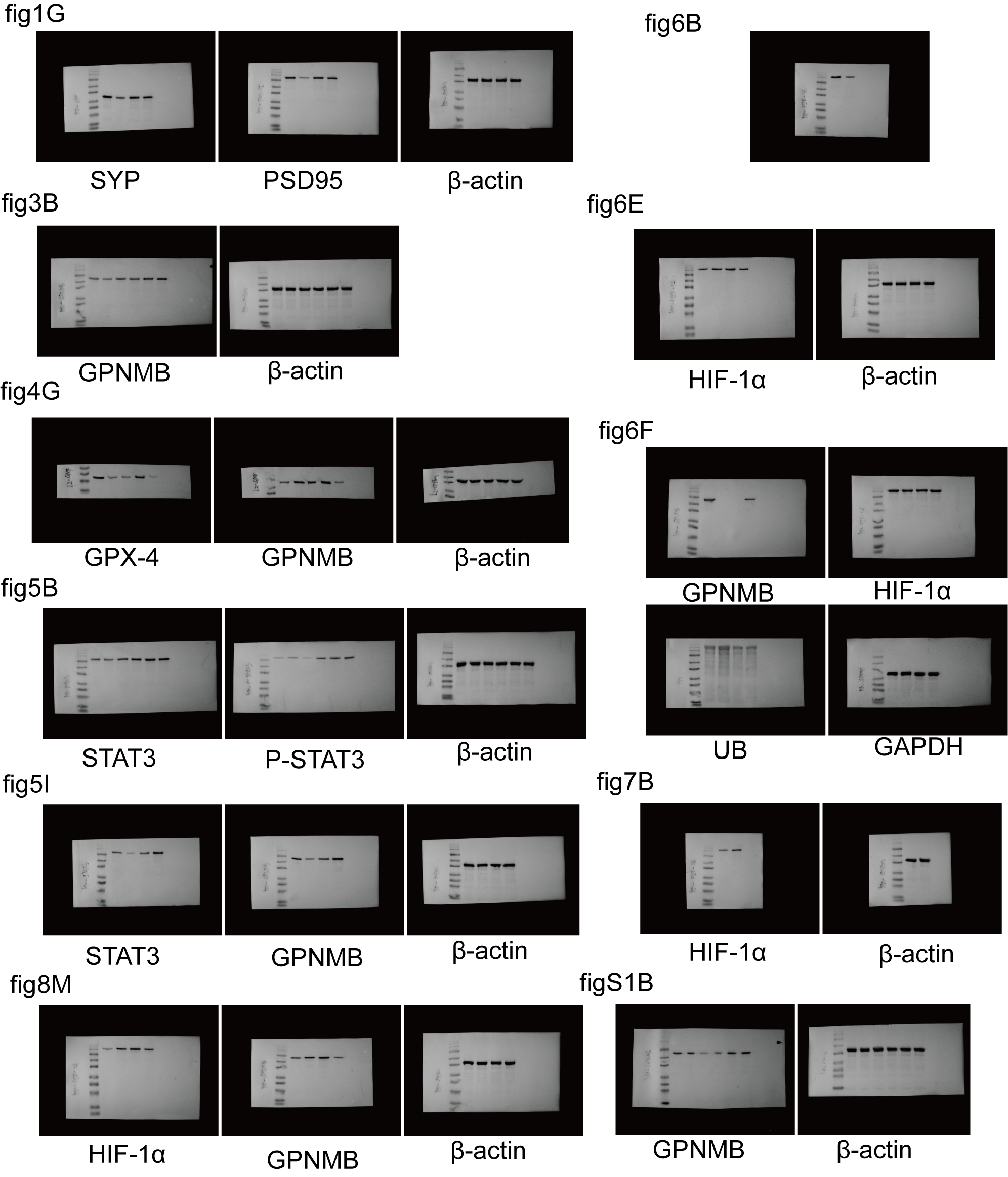

Supplement: Supplementary file 1 — Supplementary Material 1 [file 40478_2025_2069_MOESM1_ESM.tif]
